# Long-lasting, subtype-specific regulation of somatostatin interneurons during sensory learning

**DOI:** 10.1126/sciadv.adt8956

**Published:** 2025-08-15

**Authors:** Matthew B. Mosso, Mo Zhu, Xiaoyang Ma, Eunsol Park, Alison L. Barth

**Affiliations:** ^1^ Department of Biological Sciences, Carnegie Mellon University, Pittsburgh, PA 15213, USA.; ^2^ Center for the Neural Basis of Cognition, Carnegie Mellon University, Pittsburgh, PA 15213, USA.

## Abstract

Somatostatin (SST)–expressing inhibitory neurons are a major class of neocortical γ-aminobutyric acid neurons, where morphological, electrophysiological, and transcriptomic analyses indicate more than a dozen different subtypes. However, whether this diversity is related to specific roles in cortical computations and plasticity remains unclear. Here, we identify learning-dependent, subtype-specific plasticity in layer 2/3 SST neurons of the mouse somatosensory cortex. Martinotti-type, SST neurons expressing calbindin-2 show a selective decrease in excitatory synaptic input and stimulus-evoked calcium responses, as mice learn a stimulus-reward association. Using these insights, we develop a label-free classifier using basal activity from in vivo imaging that accurately predicts learning-associated response plasticity. Our data indicate that molecularly defined SST neuron subtypes play specific and highly regulated roles in sensory information processing and learning.

## Teaser

Inhibitory neuron subtypes in sensory cortex are uniquely modified by sensory-reward learning.

## INTRODUCTION

Single-cell transcriptomic analyses have redefined the complexity of neocortical networks, where more than a hundred molecularly distinct cell types have been identified (*1*). The functional relevance of these cell types in cortical computations and plasticity is largely unexplored. In particular, somatostatin (SST)–expressing γ-aminobutyric acid (GABA) neurons in the neocortex are highly heterogeneous, where studies have proposed an increasing number of distinct cell types (from 6 in 2016 to nearly 40 in 2023) (*2*). Because SST neurons are one of three major classes of cortical interneurons, densely wired into the cortical network, with robust responses that are modulated by both sensory input and cognitive variables, the functional diversity of this class is of particular interest.

In sensory neocortex, SST neurons exhibit broad receptive fields (*3*) and may encode cognitive information such as mismatch detection and attention (*4*–*6*). Dynamic modulation of layer 2/3 (L2/3) SST Ca^++^ responses during task performance has been reported in both sensory and motor cortex (*7*–*9*), and SST neuron activity has been implicated in some forms of learning (*10*, *11*). Heterogeneous SST responses, even in superficial layers, have confounded efforts to detect significant changes in population activity across conditions (*9*, *12*–*14*). Thus, mechanisms driving potential response plasticity and whether this plasticity might be differentially expressed across transcriptionally distinct subtypes of SST neurons are unclear. Without this information, it is difficult to advance hypotheses about the role of SST neurons in cortical computations and learning.

## RESULTS

### Stimulus-reward coupling drives SST response plasticity in vivo

**Fig. 1.**
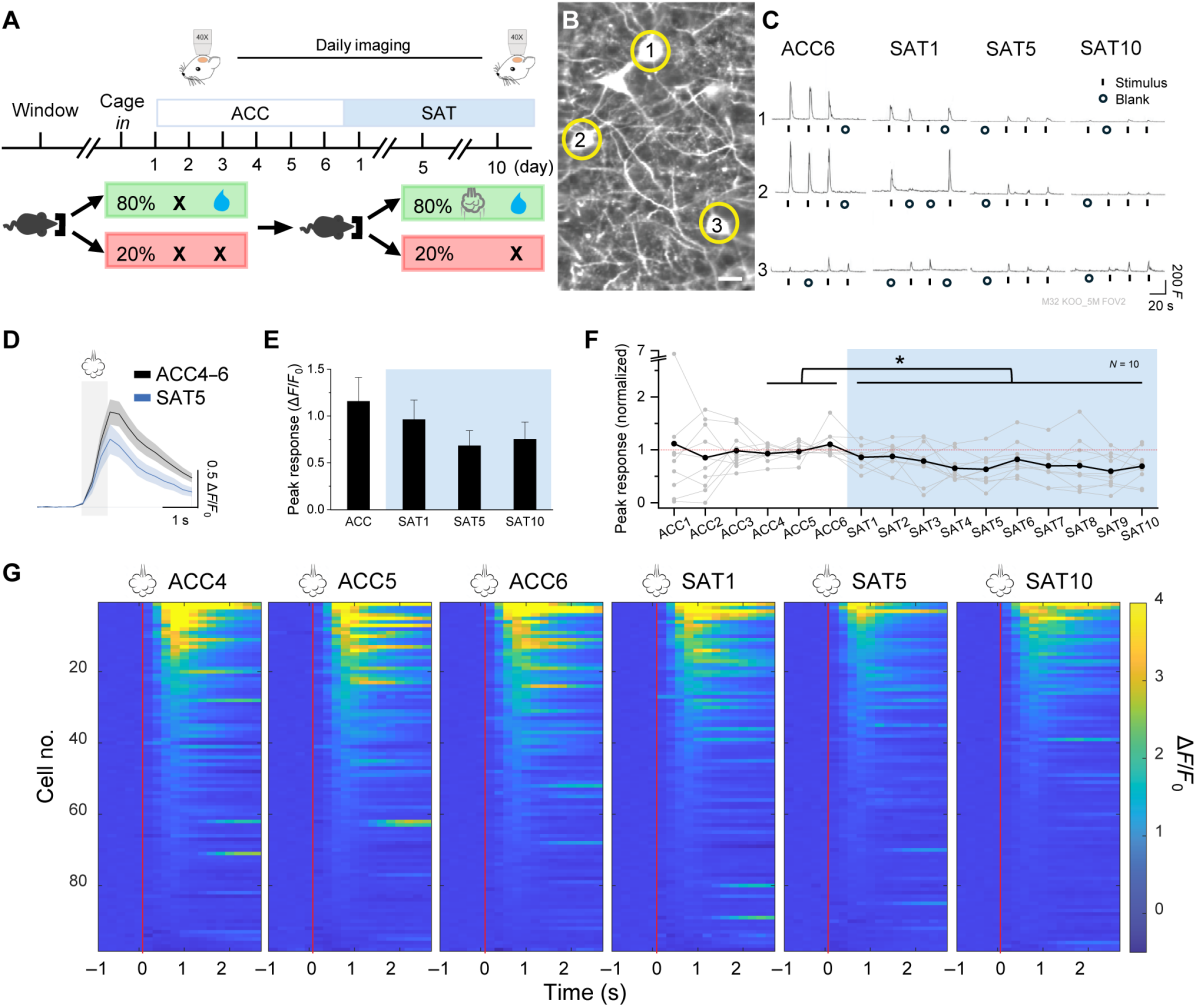
SAT reduces stimulus-evoked responses in SST neurons. (**A**) Top: Experimental timeline for longitudinal calcium imaging. Bottom: Structure of SAT. (**B**) An example imaging field of view (FOV). Scale bar, 20 μm. (**C**) Example traces of air puff–evoked responses from the same example cells on ACC6, SAT1, SAT5, and SAT10. Fluorescence (*F*) is reported in arbitrary units. (**D**) Means ± SEM air puff–evoked response during ACC4 to ACC6 and SAT5. Animal average, *N* = 10 mice. Gray shade indicates the air puff period. (**E**) Mean of evoked response peak on ACC4 to ACC6, SAT1, SAT5, and SAT10 by mice. Paired *t* test with Bonferroni correction, comparing ACC4 to ACC6 with training days, *P* = 0.18, 0.061, and 0.25 for SAT1, SAT5, and SAT10, respectively. (**F**) Peak air puff–evoked response across the ACC and training period, averaged across mice and normalized to ACC4 to ACC6 (black; *n* = 98 cells in 10 mice). Means ± SEM are shown in the figure. Gray lines indicate mean cell response for individual mice. One-way repeated-measures ANOVA, *P = 2.3 × 10^−5^. (**G**) Heatmaps of the evoked response traces of all cells rank ordered within individual imaging days. Red lines indicate air puff onset.

We initially sought to investigate long-lasting changes in SST neurons that were associated with learning, using a home-cage, freely moving training paradigm where the contingency of sensory stimuli and reward outcomes could be easily manipulated in a whisker-dependent learning task (*15*). For longitudinal in vivo imaging of neural activity, GCaMP6f was expressed in SST neurons in SST-Cre × Ai148 transgenic mice, and a cranial window was implanted over primary somatosensory (barrel) cortex (Fig. 1A). Animals were acclimated to the home-cage training environment for 6 days before training onset, where they could freely initiate trials for water that was delivered at a recessed lick port without any predictive cue (80% probability). During sensory association training (SAT), a gentle multiwhisker stimulus [4- to 6-psi (28- to 41-kPa) air puff] preceded water delivery for 80% of trials, and 20% of trials had no stimulus and no reward (blank trials; Fig. 1A and fig. S1). Learning was assessed by comparing the frequency of anticipatory licking (300 ms before water delivery) to stimulus trials and blank trials, and most of animals showed an increase in anticipatory licking to stimulus trials after 1 to 2 days of training (fig. S1). Stimulus-evoked Ca^++^ transients in SST neurons were imaged in head-fixed animals outside of the training context to avoid confounds introduced by motivational state, licking and other goal-directed movements, and cognitive variables related to performance accuracy.

**Fig. 2.**
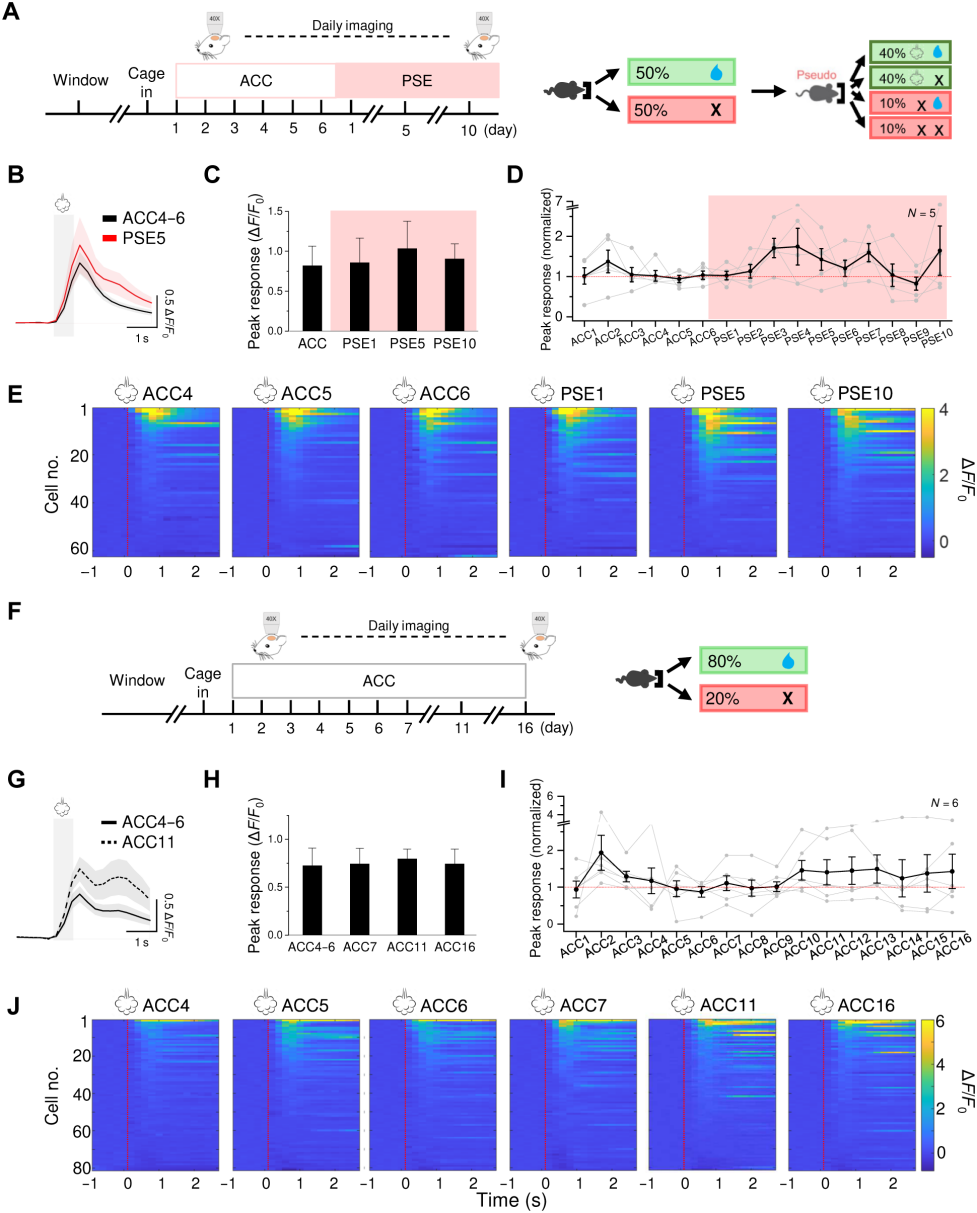
PSE and prolonged ACC did not reduce stimulus-evoked responses in SST neurons. (**A**) Left: Experimental timeline for longitudinal calcium imaging. Right: Structure of PSE. (**B**) Averaged trace of the air puff–evoked response on ACC4 to ACC6 and PSE5. Trace was averaged across five mice, 62 cells. Means ± SEM are shown in the figure. Gray shade indicates the air puff period. (**C**) Peak evoked response on ACC4 to ACC6, PSE1, PSE5, and PSE10, averaged by animal. Paired *t* test with Bonferroni correction, comparing ACC4 to ACC6 with training days, *P* = 0.76, 0.38, and 0.66 for PSE1, PSE5, and PSE10, respectively. (**D**) Peak air puff–evoked response across the ACC and training period, averaged across mice and normalized to ACC4 to ACC6 (black; *n* = 5 mice). Means ± SEM are shown in the figure. Gray lines indicate mean cell response for individual mice. One-way repeated-measures ANOVA, *P* = 0.074. (**E**) Heatmaps of the evoked response traces of all cells rank ordered within individual imaging days. Red dotted lines indicate air puff onset. (**F**) Left: Experimental timeline for longitudinal calcium imaging. Right: Structure of prolonged ACC. (**G**) Averaged trace of the air puff–evoked response on ACC4 to ACC6 and ACC11. Trace was averaged across six mice, 81 cells. Means ± SEM are shown in the figure. Gray shade indicates the air puff period. (**H**) Peak evoked response on ACC4 to ACC6, ACC7, ACC11, and ACC16, averaged by animal. Paired *t* test with Bonferroni correction, comparing ACC4 to ACC6 with prolonged ACC days, *P* = 0.27, 0.22, and 0.29 for ACC7, ACC11, and ACC16, respectively. (**I**) Peak air puff–evoked response across the ACC period, averaged across mice and normalized to ACC4 to ACC6 (black; *n* = 6 mice). Means ± SEM are shown in the figure. Gray lines indicate mean cell response for individual mice. One-way repeated-measures ANOVA (ACC4 to ACC6 versus ACC7 to ACC16), *P* = 0.17. (**J**) Heatmaps of the evoked response traces of all cells rank ordered within individual imaging days. Red dotted lines indicate air puff onset.

Daily imaging of L2/3 SST neurons in barrel cortex across 6 days of pretraining acclimation (ACC) to the training environment and 10 days of SAT revealed a progressive reduction in mean sensory-evoked Ca^++^ activity that was initiated at the onset of training {Fig. 1, B to G; animal average Δ*F*/*F*_0_: ACC (1.2 ± 0.3) versus 1-day training (SAT1) (0.96 ± 0.2), SAT5 (0.68 ± 0.2), and SAT10 (0.75 ± 0.2); *n* = 98 cells in 10 mice; ACC4 to ACC6 versus SAT1 to SAT10 [*P* = 2 × 10^−5^ by analysis of variance (ANOVA)]; see fig. S2 for histological verification of imaging sites}. This response suppression was not observed in barrel cortex from animals subjected to pseudotraining (PSE) where stimulus probability (80% of trials) was preserved but uncoupled to reward; SST responses were modestly elevated, an increase that was not significant [Fig. 2; animal average Δ*F*/*F*_0_: ACC (0.82 ± 0.3) versus PSE1 (1.0 ± 0.1), PSE5 (1.4 ± 0.3), and PSE10 (1.6 ± 0.6); *n* = 62 cells in five mice; ACC4 to ACC6 versus PSE1 to PSE10 (*P* = 0.074 by ANOVA); see fig. S2 for histological verification]. A reduction in SST responses could not be attributed to gradual response habituation during daily imaging sessions, since animals undergoing extended ACC without training showed no change (Fig. 2, G to J). No training-induced changes in SST neurons were observed in higher-order cortical areas (fig. S4), and no sex differences were observed for either group (fig. S5).

Long-lasting changes in SST activity in somatosensory cortex suggest that information-encoding circuits during learning might be saturated by our training paradigm. To investigate this, after 10 days of SAT, we returned the animals to their home cage where training was discontinued. SST population activity returned to baseline levels, at least after several weeks of housing in conventional animal caging (Δ*F*/*F*_0_: ACC6, 0.36 + 0.12; SAT10, 0.19 + 0.06; posttraining home cage, 0.52 + 0.14; *n* = 29 cells in three mice; *P* = 0.12 by ANOVA). Thus, SST activity suppression initiated by sensory learning may require sustained training to be maintained.

Because the same SST neurons could be identified across days, response profiles for individual neurons could be aligned across the imaging period (fig. S6). Daily tracking of individual SST neurons revealed that responses to sensory stimulation were highly heterogeneous (100-fold variation between neurons). Furthermore, response plasticity during SAT was also diverse, with some SST neurons decreasing and others increasing stimulus-evoked Ca^++^ transients. These properties were characteristic of individual neurons and were also maintained across the training period (fig. S6). Although heterogeneous response properties of SST neurons in superficial layers of sensory and motor cortex have been previously described (*8*, *9*, *16*, *17*), it has not been easy to connect these dynamic features to the molecular phenotype of putative SST subtypes [but see (*18*)]. The expansion of SST neuronal subtypes defined by gene expression (*1*) has suggested functional differentiation, but evidence for this has been limited (*19*).

### Anatomical synaptic analysis reveals subtype-specific SST plasticity

**Fig. 3.**
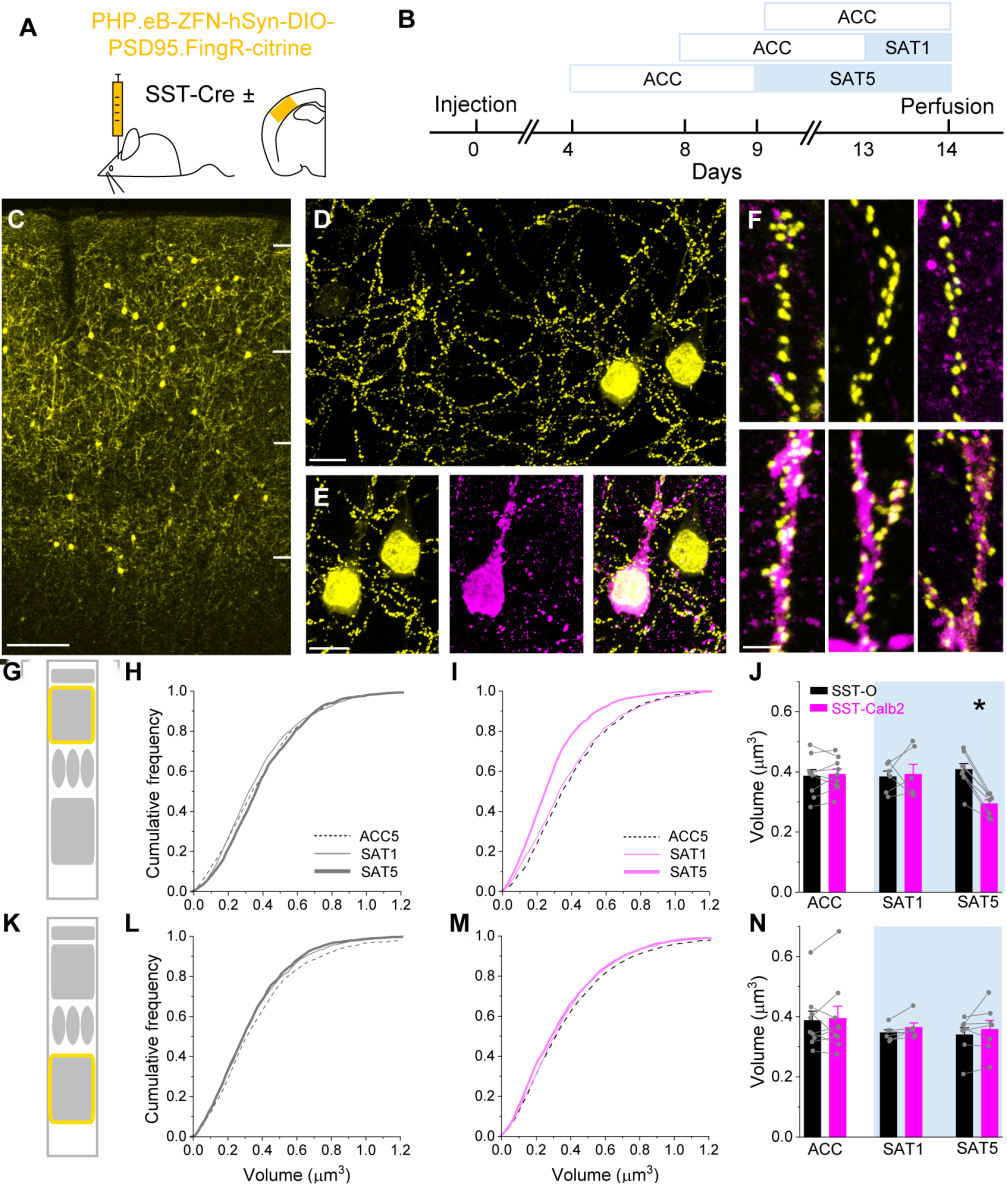
Excitatory synapses in SST-Calb2 neurons are selectively suppressed after 5 days of SAT. (**A**) Cre-dependent–PSD95.FingR-citrine was injected in barrel cortex of SST-Cre mice. (**B**) Schematic outlining injection and training timeline. Animals in fixed tissue analysis were time matched to control for viral expression time. (**C**) Image (10×) of SST-Cre mice expressing PSD95.FingR across the cortical column. Scale bar, 100 μm. (**D**) Volumetric image (63×) of PSD95.FingR-labeled SST neurons. Scale bar, 10 μm. (**E**) Immunohistochemical labeling of SST neurons (left) and PSD95.FingR-citrine (middle) calretinin expression (protein product of Calb2 gene) Scale bar, 10 μm. Right: Merged channels. (**F**) Zoomed images of PSD95.FingR puncta along dendrites from different training conditions and SST subtypes. Top: Calb2-negative dendrites. Bottom: Calb2-positive dendrites. Left to right: ACC, SAT1, and SAT5. Scale bar, 3 μm. (**G**) Schematic indicating layer imaging FOV was captured (L2/3). (**H**) Cumulative distribution of PSD95 puncta volume in L2/3 SST-O neurons. (**I**) Same as (H) for L2/3 SST-Calb2 neurons. (**J**) Within animal comparison of mean PSD95 puncta volume between SST-O (black bars) and SST-Calb2 (magenta bars) after experiencing ACC and 1 or 5 days of SAT. *P = 8 × 10^−4^. (**K** to **N**) Same as (G) to (J) but for L5 SST-O and SST-Calb2 SST neurons.

We hypothesized that the suppression of SST sensory-evoked responses, particularly as observed outside of the training context, might be linked to long-lasting changes in SST neurons during SAT (*15*, *20*). Intrinsic excitability of SST neurons was similar before and after training (fig. S7). Because they are densely connected into the local network of pyramidal neurons (*21*), we hypothesized that their decreased activity might be related to a reduction in excitatory synaptic drive. For quantitative analysis of excitatory synaptic input in fixed tissue, a Cre-dependent PSD95.FingR intrabody (*20*) tagged with mCitrine was virally transduced in barrel cortex for SST synaptic labeling (Fig. 3, A to E). Synaptic PSD95 levels are directly correlated with excitatory synaptic strength and are bidirectionally modified during synaptic potentiation or depression (*22*, *23*). Thus, tracking synaptic PSD95 can yield insight into bidirectional excitatory plasticity. Automated detection and volume analysis of thousands of PSD95 puncta from L2/3 SST neurons (fig. S8) showed a small but significant population decrease in volume between ACC and SAT conditions, particularly at later stages of training (fig. S9, A to C).

L2/3 SST neurons in barrel cortex are anatomically and molecularly heterogeneous, composed of SST-Calb2 (Martinotti type, with a prominent axon in L1), SST-Mme (targeting mainly L2/3), and SST-Chodl neurons (long-range projecting) (*24*, *25*). Recent transcriptomic analysis suggests that there may be at least several other subtypes contained within these three subclasses (*1*). We hypothesized that the modest shift in PSD95 puncta size in L2/3 might be reflected in a subclass-dependent regulation of excitatory input strength. To investigate this, we immunostained PSD95.FingR SST neurons with an antibody directed against calretinin, the protein coded by the Calb2 gene (Fig. 3F). PSD95 puncta size was compared between SST-Calb2 and unlabeled SST neurons (SST-other/SST-O) dendrites from control (ACC) and also trained mice (Fig. 3, G to J and K to N). In ACC mice, a comparison of PSD95 puncta in SST-Calb2 and SST-O dendrites showed no difference in puncta volume distribution [Fig. 3, H to J; ACC SST-O (0.39 ± 0.02) versus SST-Calb2 (0.39 ± 0.02 μm^3^), *n* = 1800 puncta in nine mice]. However, 5 days of SAT drove a prominent and highly significant reduction in puncta volume that was restricted to L2/3 SST-Calb2 dendrites [Fig. 3, H to J; SST-O (0.41 ± 0.02) versus SST-Calb2 (0.29 ± 0.01 μm^3^), *n* = 1600 puncta in eight mice; paired *t* test, *P* = 8 × 10^−4^]. This difference was observed when averaged across animals and also when comparing adjacent SST-Calb2 and SST-O cells within the same field of view (FOV), where imaging conditions were matched (Fig. 3J).

Because analysis was carried out in fixed tissue, we could also examine SAT-associated changes in PSD95 puncta size for SST-Calb2 cells in deeper layers. However, we did not observe a reduction in PSD95 puncta volume in L5, either overall (fig. S9, G to I) or for SST-Calb2 neurons in particular [Fig. 3, K to N; SAT5 SST-O (0.34 ± 0.02) versus SST-Calb2 (0.36 ± 0.03 μm^3^)]. Notably, a reduction in PSD95 puncta volume, either for SST-Calb2 or for SST-O neurons, was not evoked by PSE where whisker stimulation had no value in predicting water delivery (fig. S10, A to H). These effects were not observed in primary visual cortex where SST-Calb2 neurons showed no change in puncta size after 5 days of SAT (fig. S10, A to C).

Last, to test whether active sensation during exploratory behaviors could be sufficient to induce changes in PSD95 puncta volume in SST-Calb2 neurons, we housed mice in an enriched environment for multiple days (fig. S11, I and J). However, this treatment also failed to modify either overall PSD95 puncta volume between SST-O and SST-Calb2 neurons or the relative difference between SST-Calb2 and SST-O neurons within barrel cortex tissue from the same animal. Thus, the pairing of fully predictive whisker stimulation and water reward during SAT uniquely drove a long-lasting reduction in excitatory input onto SST-Calb2 neurons.

The relative timing of depressed stimulus-evoked SST activity (early during training) and a reduction in puncta volume (later in training) suggested that decreased SST neuron activity might initiate their postsynaptic reduction in PSD95 puncta volume. To test this hypothesis, we used chemogenetic suppression of activity by virally transducing the inhibitory designer receptors exclusive activated by designer drugs (DREADDs) receptor hM4Di into SST neurons in barrel cortex, in the absence of any training paradigm (fig. S12). Five days of clozapine-*N*-oxide (CNO) administration was sufficient to drive a reduction in PSD95 puncta in hM4Di-expressing SST neurons compared to unlabeled neurons in the same L2/3 FOV, for both SST-O and SST-Calb2 cells [fig. S12, C to H: SST-O comparison for hM4Di^+^ (0.29 ± 0.01) versus nonexpressing SST-O (0.38 ± 0.03 μm^3^), *n* = 1200 puncta in six mice each; paired *t* test, *P* = 0.01; fig. S13, I to N: SST-Calb2 comparison for hM4Di^+^ (0.23 ± 0.01) versus nonexpressing SST-Calb2 (0.36 ± 0.02 μm^3^), *n* = 1200 puncta in six mice each; paired *t* test, *P* = 0.004]. Thus, chemogenetic suppression of activity in the broader population of L2/3 SST neurons could phenocopy the effects of SAT on PSD95 puncta size. The selective reduction in excitatory inputs onto SST-Calb2 neurons during SAT suggests that these neurons are specifically engaged by neural circuits related to learning.

### SST-Calb2 neurons show selective response suppression during SAT

**Fig. 4.**
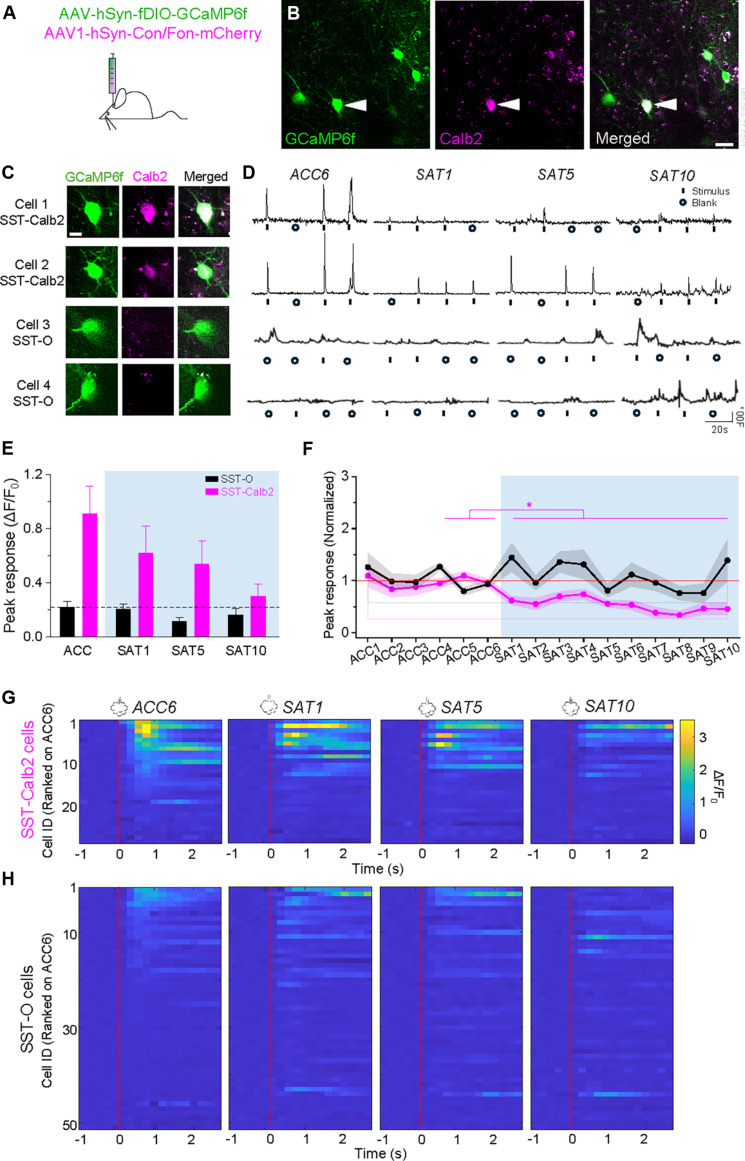
SAT specifically reduced evoked response in SST-Calb2 neurons. (**A**) Schematic demonstrating the stereotaxic delivery of viral constructs into mouse barrel cortex. (**B**) Example FOV expressing GCaMP6f in SST cells and mCherry in Calb2-positive cells. Scale bar, 20 μm. (**C**) Left: Example SST-Calb2 and SST-O neurons showing GCaMP6f expression (green) and Calb2 expression (magenta), with merged images (right) displaying both markers. Scale bar, 10 μm. (**D**) Example traces of example SST-Calb2 and SST-O cells on ACC6, SAT1, SAT5, and SAT10. (**E**) Mean peak response across ACC1 to ACC6 and SAT1 to SAT10. The magenta lines represent the average peak response across 28 SST-Calb2 cells, while the black lines represent the average peak response across 51 SST-O cells collected in seven mice. One-way repeated-measures ANOVA, *P* = 1.3 × 10^−12^ for SST-Calb2 and *P* = 0.50 for SST-O. (**F**) Mean peak response of SST-Calb2 and SST-O cells on ACC4 to ACC6, SAT1, SAT5, and SAT10. Paired *t* test with Bonferroni correction, comparing between SST-Calb2 and SST-O cells within different imaging days, *P = 5.5 × 10^−5^, 0.10, 0.0018, and 0.12 on ACC4 to ACC6, SAT1, SAT5, and SAT10, respectively. (**G**) Response heatmaps of SST-Calb2 rank ordered on ACC6. Red line indicates in the onset of air puff. (**H**) As in (G), but for SST-O cells.

Are SST-Calb2 neurons responsible for the population decrease in sensory-evoked activity in L2/3 SST neurons observed during in vivo Ca^++^ imaging? *Calb2* is also expressed in a subpopulation of vasoactive intestinal peptide (VIP) interneurons, complicating efforts to monitor these neurons without intersectional genetics. Thus, we generated *SST-Flp* × *Calb2-Cre* mice with viral transduction of SST neurons using an Flp-dependent GCaMP6f and a Cre-dependent mCherry construct for detection of Calb2 neurons (Fig. 4, A and B). Overlap in GCaMP6f and mCherry signal was an indicator of SST-Calb2 neurons (fig. S13), although *Calb2* is expressed either transiently or at low levels in other L2/3 SST subtypes (*24*, *25*), and post hoc immunolabeling of Calb2 in SST neurons detects only ~75% of neurons labeled through intersectional genetics (*26*) or viral transduction (fig. S14), indicating that *Calb2* gene expression is not a precise indicator of protein levels. In general, Ca^++^ signals using virally transduced GCaMP6f expression were weaker than observed in the transgenic Ai148 line, but both spontaneous and stimulus-evoked activity could be clearly distinguished.

On the basis of decreased PSD95 puncta volume in our fixed-tissue analysis, we predicted that SST-Calb2 neurons in barrel cortex would show a reduction in stimulus-evoked activity across the SAT period (see fig. S15 for behavior and fig. S16 for histological verification). This was indeed the case (Fig. 4, C to H). SST-Calb2 neurons showed a significant reduction in peak amplitude of the whisker-evoked response that progressively decreased over training (SST-Calb2 fold change: SAT1, 0.62 ± 0.083; SAT5, 0.56 ± 0.068; SAT10, 0.46 ± 0.12; *n* = 28 cells in seven mice; ACC4 to ACC6 versus SAT1 to SAT10, *P* = 1.3 × 10^−12^ by ANOVA). In comparison, unlabeled cells (which could represent SST-O neurons or SST-Calb2 neurons that were not virally transduced by the mCherry tag, designated for clarity as SST-O) showed no significant decrease in sensory-evoked activity (SST-O fold change: SAT1, 1.4 ± 0.27; SAT5, 0.81 ± 0.12; SAT10, 1.4 ± 0.40; *n* = 51 cells in seven mice; ACC4 to ACC6 versus SAT1 to SAT10, *P* = 0.5 by ANOVA). Consistent with results from Fig. 2, PSE did not reduce stimulus-evoked responses, in either SST-Calb2 or SST-O neurons (fig. S17, E and F). These data suggest that sensory-evoked activity in SST-Calb2 neurons is selectively depressed by SAT and initiated before changes in excitatory input.

### Predictive classification of SST-Calb2 plasticity using in vivo Ca^++^ signals

**Fig. 5.**
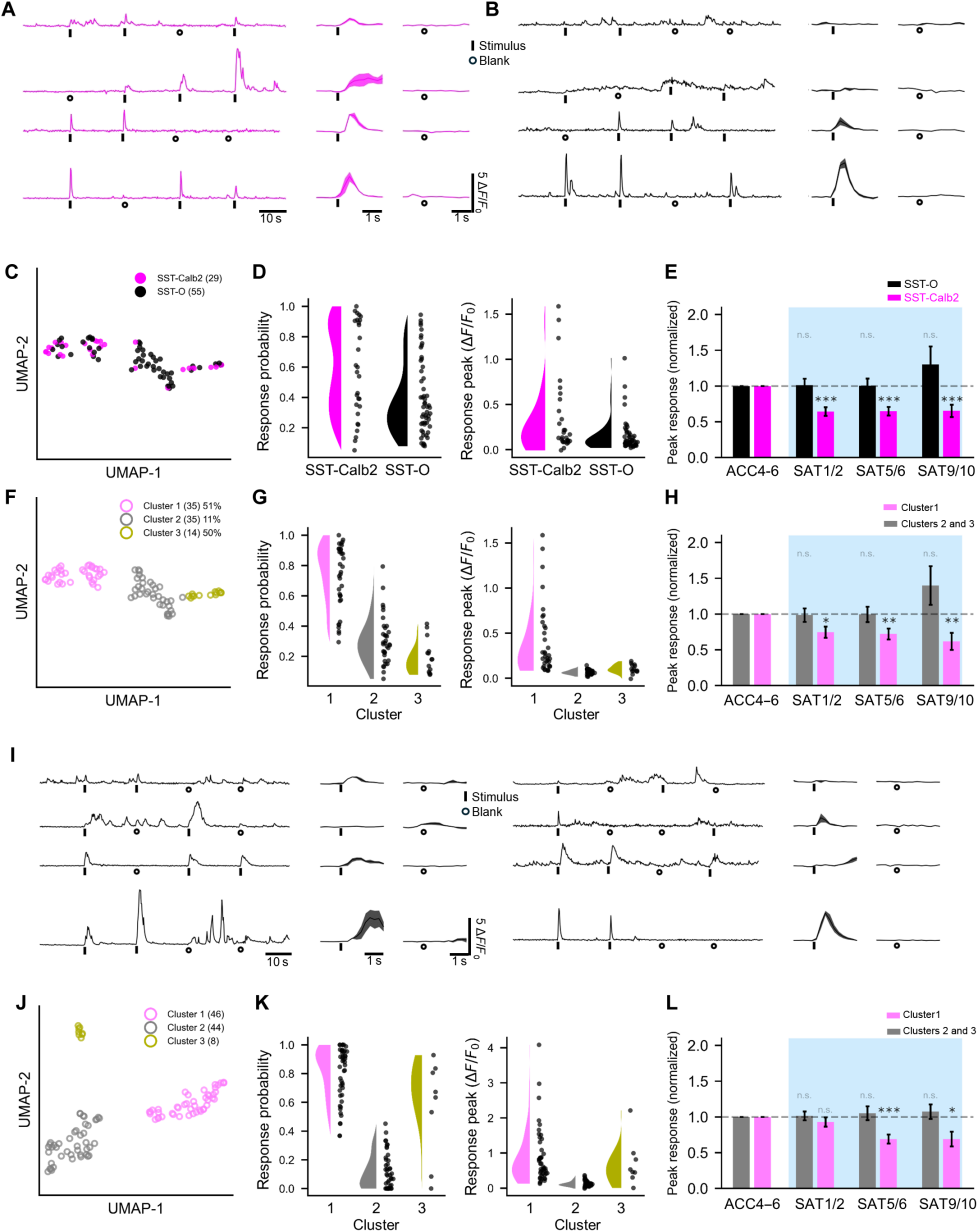
Cluster-based SST classification from pretraining activity predicts response plasticity during learning. (**A**) Example Ca^++^ transients during pretraining (ACC) from SST-Calb2 neurons identified in Fig. 4. (**B**) As in A_1_ but for SST-O neurons. (**C**) Uniform Manifold Approximation and Projection (UMAP) visualization of SST clustering using ACC4 to ACC6 response properties (*n* = 84 cells in seven animals). (**D**) Comparison of stimulus-evoked response probability and response peak for genetically labeled SST-Calb2 and SST-O neurons. (**E**) Mean peak stimulus-evoked response (all trials) of SST-Calb2 neurons during SAT, normalized to ACC4 to ACC6. Data were averaged for SAT1/2, SAT5/6, and SAT9/10. Paired *t* test with Bonferroni correction, comparing ACC4 to ACC6 with training days, ***P = 7.9 × 10^−6^, 1.1 × 10^−5^, and 1.5 × 10^−4^ for SAT1/2, SAT5/6, and SAT9/10 (unpaired *t* test due to lost data), respectively. n.s., not significant. (**F**) Cluster assignment of neurons from (B) for putative SST-Calb2 (cluster 1, 51% SST-Calb2 cells) and putative SST-O neurons (cluster 2, 11%; cluster 3, 50% SST-Calb2 cells). (**G**) As in (C) but for the assigned clusters. (**H**) As in (D), but for cluster 1 (putative SST-Calb2) versus clusters 2 and 3 (combined for comparison due to the small number of cluster 3 cells). Paired *t* test with Bonferroni correction, comparing ACC4 to ACC6 with training days, *P = 0.010, **P = 0.003, and 0.002 for SAT1/2, SAT5/6, and SAT9/10 (unpaired *t* test due to lost data), respectively. (**I**) Example Ca^++^ transients from SST neurons during pretraining (ACC5) from the unlabeled, SST-Cre × Ai148 dataset in Fig. 1. (**J**) As in (E), but for ACC data from the unlabeled dataset (*n* = 98 cells from 10 animals). The putative SST-Calb2 group (cluster 1) and putative SST-O (clusters 2 and 3) are indicated. (**K**) As in (F), but for clusters identified in (I). (**L**) As in (G), but for cluster 1 versus clusters 2 and 3 from the unlabeled dataset. Paired *t* test with Bonferroni correction for ACC4 to ACC6 for cluster 1 versus training days, *P* = 0.843, ***P = 4.8 × 10^−5^, and **P = 0.014 for SAT1/2, SAT5/6, and SAT9/10, respectively.

Sensory-evoked activity in SST neurons is highly heterogeneous even under basal, home-cage conditions without training (*13*, *16*). We observed that during ACC, SST-Calb2 neurons identified by mCherry expression typically showed a higher sensory-evoked response compared to unlabeled SST neurons (Figs. 4, G and H, and 5 and fig. S18). These data suggested that the SST-Calb2 subset of neurons might be defined by activity properties, irrespective of their plasticity during learning. Using 30 features (of 117 total) extracted from stimulus-evoked and spontaneous activity in this dataset (mainly related to response probability and peak stimulus-evoked responses over different time intervals; figs. S19 and S20), we applied a clustering-informed heuristic classification to distinguish SST-Calb2 from SST-O neurons (Fig. 5E). We used silhouette scoring, inertia, and eigenvalues to determine that three clusters were optimal for this dataset (fig. S21), a finding that is consistent with three transcriptomic classes localized to L2/3 (*25*, *26*). This heuristic clustering did not exclusively assign SST-Calb2 neurons to one group (Fig. 5, B and E); however, we reasoned that low levels of Calb2 expression in other SST subclasses (*1*) and also variability in viral uptake might account for this distribution.

Using this computational grouping, we manually assigned all SST neurons in one cluster (cluster 1) as putative SST-Calb2 neurons and combined the other clusters into a single group (clusters 2 and 3; Fig. 5G). We then asked whether SAT-dependent response suppression would be concentrated in cluster 1. Clustering based on SST activity properties before training (during ACC) showed strong predictive value in identifying neurons with SAT-dependent response suppression (Fig. 5G). Mean peak response from early (SAT1/2), mid (SAT4/5), and late (SAT9/10) time points was significantly reduced for cluster 1 (putative SST-Calb2–classified neurons) but not for clusters 2 and 3 [Fig. 5G; cluster 1, fold change versus ACC4 to ACC6 for SAT1/2 (0.75 ± 0.08), SAT5/6 (0.72 ± 0.08), and SAT9/10 (0.62 ± 0.1), *n* = 35 cells; clusters 2 and 3, fold change versus ACC4 to ACC6 for SAT1/2 (0.98 ± 0.09), SAT5/6 (1.0 ± 0.1), and SAT9/10 (1.4 ± 0.3), *n* = 49 cells]. Thus, identification of SAT-induced PSD95 puncta plasticity that was concentrated in SST-Calb2 neurons subsequently enabled us to identify unique activity features of SST subtypes from pretraining ACC data that could predict response plasticity during learning.

Intersectional genetic methods for cell type identification are experimentally cumbersome and can suffer from a high false-positive rate due to low levels of marker gene expression in other cell classes, particularly true for the *Calb2* gene (*26*). Label-free cell clustering using features of neural activity would be a major advance in deciphering cell identity from large-scale imaging or recording experiments. Thus, we asked whether this classifier might enable us to identify putative SST-Calb2 neurons from the larger unlabeled dataset, that is, neurons from SST-Cre transgenic mice (*n* = 98 cells in 10 mice). Neurons with transgenic expression of GCaMP6f were typically brighter than virally labeled cells, complicating application of a fixed-parameter classifier that would not generalize between datasets, a potential problem that was addressed using clustering-informed classification. Again, classification was carried out using data only from the pretraining ACC period.

Similar to results from the labeled SST-Calb2 dataset in Fig. 5E, cluster analysis segregated SST neurons into multiple groups, one of which was assigned as cluster 1 (putative SST-Calb2) based on similarity of their response properties to the SST-Calb2 cells (Fig. 5, I and J). Again, we used this grouping to ask whether cluster 1 would selectively show response suppression during learning. Clustering of SST groups using responses from the ACC period accurately predicted response suppression during SAT, where cluster 1 showed a highly significant, 30% response suppression during SAT, similar to results from Figs. 4E and 5 (D and G) [cluster 1, fold change versus ACC4 to ACC6 for SAT1/2 (0.93 ± 0.06), SAT5/6 (0.69 ± 0.06), and SAT9/10 (0.69 ± 0.1), *n* = 46 cells]. In comparison, SST neurons in clusters 2 and 3 showed no significant change during SAT [clusters 2 and 3, fold change versus ACC4 to ACC6 for SAT1/2 (1.01 ± 0.06), SAT5/6 (1.05 ± 0.1), and SAT9/10 (1.07 ± 0.1), *n* = 52 cells].

PSE did not depress stimulus-evoked SST activity in superficial layers of barrel cortex; responses were increased in at least a subset of neurons (Fig. 2E). We verified our clustering approach on identified SST-Calb2 neurons from pseudotrained animals in fig. S17, using the same pretraining features as for the SAT animals. As expected from in vivo imaging experiments shown in Fig. 2 and fig. S17, PSE did not show a reduction in sensory-evoked responses for cluster 1 (putative SST-Calb2) versus cluster 2 and cluster 3 neurons (fig. S22). These data suggest that plasticity in SST-Calb2 neurons is selectively initiated by predictive sensory-reward information but not when sensory inputs are unrelated to reward outcome.

## DISCUSSION

Developing a census of molecularly and functionally defined neuronal cell types is an important step in understanding and replicating the computational properties of the neocortex, a six-layered anatomical structure that is highly conserved across cortical areas and mammalian species (*27*). Although cortical lamina provides a useful structure to understand cortical information flow (*28*), the diversity of neuronal subtypes and their connectivity even within a layer has complicated this model (*25*, *29*). Transcriptomic studies indicate that there may be hundreds of distinct cell types in the neocortex, with substantial diversity even within defined subclasses of excitatory and inhibitory neurons (*13*). The correspondence of this transcriptomic diversity with neural activity and function remains poorly understood, particularly for cortical interneurons.

Although functional diversity in neocortical SST neurons has been suggested by recent studies (*9*, *19*, *30*), linking this diversity to molecularly defined subclasses using transcriptomic approaches has been elusive (*18*). Our in vivo imaging and anatomical analysis indicate that SST-Calb2 Martinotti neurons in superficial layers of sensory cortex are selectively modified by stimulus-reward association during learning but not in PSE, when whisker stimuli have no predictive value for water delivery. Thus, L2/3 SST-Calb2 neurons lie in a distinct node of the cortical circuit responsive to contingency information and are likely to be distinguished by their synaptic connectivity and/or selective responses to neuromodulators. Furthermore, chemogenetic inhibition of SST neurons was sufficient to initiate a loss of associated PSD95 synaptic sites onto any SST neuron in superficial layers, suggesting that the SAT-dependent effect does not result from a cell type–specific transcriptional program in SST-Calb2 neurons but may be directly related to reduced activity of this subclass during training. However, because Calb2 expression is not absent from all other SST subclasses, this gene will not be a perfect predictor of cell identity and plasticity during learning.

Because SST activity was suppressed before a reduction in PSD95 puncta size during SAT, our data suggest that decreased synaptic PSD95 specifically in SST-Calb2 neurons may result from suppression of sensory-evoked activity in these cells. It will be of great interest to identify the circuits involved in SST-Calb2 activity suppression during learning. Because SST neurons receive strong inhibition from GABA VIP neurons (*31*), changes in VIP activity during stimulus-reward association could be an early step in the SAT-dependent regulation of SST output.

Our experiments purposefully monitored SST activity outside of the training context, enabling us to avoid confounds from task-related activity (*32*) to focus on sensory-evoked responses. Because we also observed marked synaptic changes onto SST-Calb2 neurons that were preserved in fixed tissue, changes in the stimulus-evoked responses of SST neurons are likely to be present during training and will dynamically influence sensory processing.

Activity-based cell type classification has been of great interest to neuroscientists, as it can provide structure to in vivo recordings that shed light on dynamic neural computations (*33*, *34*). The increasing number of molecularly defined neural subtypes in the brain has complicated these efforts. Our anatomical results and cluster-based classification data validate transcriptomic and anatomical distinctions between SST subtypes, indicating that these neurons play selective roles in cortical reorganization during learning. We used a clustering-informed classification method that could adapt to variation in Ca^++^ signals collected from different genetic methods and experimental setups. This cluster-based classification approach provides a robust and easily adopted method for label-free, cell type identification from in vivo recordings, an important step in understanding the algorithms that underlie information processing in the brain.

## MATERIALS AND METHODS

### Animals

For GCaMP6f imaging in SST neurons, we crossed Sst-IRES-Cre mice (the Jackson Laboratory, #013044) to Ai148(TIT2L-GC6f-ICL-tTA2)-D mice (the Jackson Laboratory, #030328). For GCaMP6f imaging in calretinin-expressing SST neurons, we crossed Sst-IRES-Flp mice (the Jackson Laboratory, #028579) to Cr-IRES-Cre (Calb2-IRES-Cre) mice (the Jackson Laboratory, #010774). Juvenile to adult transgenic mice (1.5 to 6 months of age) were used for cranial window surgery and virus injection. They recovered for 1 to 3 weeks before commencing 2-photon (2P) in vivo imaging. Sst-IRES-Cre mice (the Jackson Laboratory, #013044) were used for fixed tissue analysis. Male and female mice were used for all experiments and approximately balanced across control and experimental datasets. All procedures for animal experiments were performed following protocols (PROTO201600045) approved by the Institutional Animal Care and Use Committee at Carnegie Mellon University and were carried out in accordance with US National Institutes of Health guidelines.

### Cranial window surgery

Surgery was done under isoflurane anesthesia (4% for induction and 1.5 to 2% for maintenance). Mice were put on a heat pad with a temperature control system (FHC, 40-90-8D) to maintain body temperature. Eyes were covered with Puralube Vet Ointment to prevent drying. Fur was removed with Nair, and the skin was cleaned with povidone and then incised expose the skull. The skull was scraped with a dental blade (Salvin, 6900) to remove the periosteum and abraid the surface for headpost attachment. On the left hemisphere, S1 coordinates (3.5 mm lateral and 1 mm posterior to bregma) and a 3-mm-diameter circle centered at the coordinates were marked with a pen. A thin layer of tissue adhesive (3M Vetbond) was applied to the skull, and then a custom-made headpost was attached to the right hemisphere with cyanoacrylate glue and dental cement (Lang Dental, 1223PNK). With a dental drill (Dentsply, 780044), the skull was thinned along the 3-mm-diameter circle. Thinned skull was removed by lifting a spot of the thinned region with forceps. Minor bleeding was stopped with saline-soaked Gelfoam (Pfizer, 00009032301), and a glass window composed of a 3-mm-diameter glass (Warner Instruments, 64-0726) attached to a 4-mm-diameter glass (Warner Instruments, 64-0724) by ultraviolet adhesive (Norland, 717106) was applied over the craniotomy. The window was sealed with 3M Vetbond and then with cyanoacrylate glue. All exposed skull area except the window was covered with dental cement. A well surrounding the window was built with dental cement for microscopy using a water immersion lens. At the end of the surgery, ketoprofen (3 mg/kg) was injected subcutaneously, and the mouse was allowed to recover in a heated cage. Mice were given 1 to 3 weeks of recovery before imaging commenced.

### Stereotaxic injections

Male and female mice expressing Cre recombinase under the SST promoter aged postnatal day 50 (P50) to P100 were induced into anesthesia with 4% isoflurane and administered a maintenance dose of ~1.5% isoflurane throughout the duration of surgery. Using a dental drill, a burr hole was drilled preserving the final layer of skull to minimize damage to the cortex. Saline was washed over the skull to soften the injection zone before insertion of the glass pipette. Mice were injected with ~80 nl of AAV-PHP.eB-ZFN-hSyn-DIO-PSD95.FingR-Citrine-reg.WPRE into S1 (−3.5 mm lateral and −1.25 mm posterior relative to lambda) using a Nanoject (Drummond Scientific). Postinjection, sutures were used to close the scalp lesion. Following surgery, mice were singly housed to reduce possible confounds of different socialization levels among mice compared across trained or naïve conditions.

For chemogenetic experiments designed to monitor changes in PSD95 puncta after suppressing activity, we coinjected pAAV8-hSyn-DIO-hM4Di-mCherry (Addgene, #44362) with AAV-PHP.eB-ZFN-hSyn-DIO-PSD95.FingR-Citrine-reg.WPRE into barrel cortex using the stereotaxic injection procedures outlined above. Aliquots of CNO (APExBIO) made up in dimethyl sulfoxide (0.01 mg/μl) were placed in the drinking water to approximate the dosage of 1 mg/kg per day based on animal initial weight and estimated water consumption of 2 ml/day. To administer CNO, mice were placed in the training cage without any air puff cue and were freely able to collect water containing CNO for 5 days. Similar to the ACC period in other SAT training experiments, trials dispensed water at 80% probability.

For GCaMP6f imaging and calretinin labeling in SST neurons using viral methods, we injected AAV1-Ef1a-fDIO-GCaMP6f (Addgene, #128315) mixed at a 1:1 ratio with AAV8-Ef1a-Con/Fon-mCherry (Addgene, #137132) or pAAV8-hsyn-DIO-mCherry (Addgene, #50459) in Sst-IRES-Flp x Cr-IRES-Cre (Calb2-IRES-Cre) mice using a Nanoject II (Drummond Scientific) directly before application of the cranial window implant. After craniotomy, ~0.6 μl of virus (~1.84 × 10^13^ vg/ml) was injected into the barrel cortex (three sites across the cranial window, 0.3 mm below the pial surface). The virus expression period lasted 26 to 42 days before the commencement of imaging.

### Sensory association training

Mice were trained to associate a multiwhisker stimulus with a delayed water reward in an automated training cage (*15*, *35*). Mice were singly housed in a home cage connected to a freely accessible chamber with a water port and an air puff delivery tube. Animals were not water deprived. During the cage ACC period, animals could freely approach the lick port and initiate a trial by breaking an infrared beam. During the ACC period, nose pokes initiated a random delay period lasting 1.2 to 1.8 s, followed by delivery of a water droplet (~10 μl) dispensed at 80% probability (i.e., 20% of nose pokes did not result in water delivery). During SAT, 80% of beam breaks were followed by a random delay of 0.2 to 0.8 s and then a gentle air puff [6 psi (41.37 kPa), 500-ms duration] delivered to the right-side whiskers, followed 500-ms delay and then water delivery (fig. S1). This training paradigm has been described in detail elsewhere (*15*, *20*, *35*). Anticipatory licking frequency was assessed during the 300-ms period immediately before water delivery. The sensory cue was fully predictive; i.e., all air puff stimuli were followed by water. During SAT, 80% of trials consisted of the predictive air puff, followed by the water reward. The remaining 20% of trials had no stimulus and no water reward (blank trials). There was a 2-s time-out between trials, where nose pokes would not trigger water delivery. Animals were freely moving and lived in the training cages except during daily imaging periods. Mice typically carried out ~500 trials/day.

During PSE, the air puff stimulus randomly preceded either water or blank trials, so that air puff had no predictive value for water delivery. During the ACC period, water was delivered with a 50% probability to match the probability used for PSE. During PSE, air puff was delivered in 80% of the trials, but water followed the stimulus for only half the trials, while the other half of stimulus trials were followed by no water delivery (fig. S3) (*15*). To further decouple the stimulus from the reward, water was delivered without a preceding air puff for half of the remaining nonstimulus trials. Therefore, the air puff stimulus and water reward were entirely decoupled during PSE. Animal performance was calculated as described for SAT.

Both SAT and pseudotrained animals undergoing 2P imaging experienced 6 days of ACC, followed by 10 days of training. For each animal, total number of trials (water + blank trials) and anticipatory lick frequencies [licks occurring in a 300-ms window right before water delivery; see (*35*)] were calculated for every 4-hour bin using a custom MATLAB code. Any 4-hour bin with fewer than 10 trials was removed from the averaged data, since lick frequency on blank trials could not be accurately assessed from one to two trials. Performance was calculated by taking the difference between anticipatory licking frequencies (in hertz) during stimulus versus blank trials (licking_stimulus_ − licking_blank_). Absolute differences in calculated lick frequency between stimulus and blank trials for the last 20% of trials on a given day were compared using a Wilcoxon signed-rank test.

### 2P in vivo imaging

All training sessions were conducted within the automated home-cage training system, ensuring a consistent and controlled environment for behavioral learning tasks. For 2P imaging experiments, mice were removed from their home-cage environment for brief periods of 1 hour/day, typically around noon. No lick port was present during training, and the number of stimulus trials was kept to a minimum (~15) to prevent extinction. No difference in performance after imaging sessions was observed, indicating that this brief exposure did not alter the learned association (*36*). Mice were removed from the training cage around noon each day, briefly anesthetized with volatile isoflurane (4% for ~20 s) to head-fix the animal under the microscope and then allowed to recover for 3 to 5 min before imaging. Animals were awake and ambulatory on the wheel before imaging began. Imaging was carried out with 2P microscope (Femto2D Galvo), equipped with a Mai Tai laser MTEV HP 1040S (Spectra-Physics), a 4× air objective lens [Olympus UPLFLN 4× numerical aperture (NA) 0.13], and a 40× water objective lens (Olympus LUMPLFLN 40×W NA 0.8). Images were acquired with MES software v.6.1.4306 (Femtonics).

Blood vessel morphology under 4× bright field was used to find the same imaging FOV as the previous session. The pial surface (*z* = 0) was defined as the plane just below the dura mater that looks like a textured membrane under 40× bright field. In 40× 2P mode, the *x*, *y*, and *z* positions of the neurons were aligned to match the previous session image. A 950-nm excitation was used to image GCaMP6f signals, and emission fluorescence was detected with photomultiplier tube (PMT; Hamamatsu, H11706P-40). Laser power and PMT voltage were kept constant for each animal across its imaging sessions. Images were acquired at 5.11 Hz with ~270 μm–by–300 μm FOV and a resolution of 0.7 μm per pixel. Imaging depth was ~200 μm below pia (L2/3), and one to two FOVs were imaged per mouse.

For each day, ~3 to 5 min after head fixation, one or two 10-min imaging sessions were carried out, with a 1-min break in between. At the beginning of each imaging session, spontaneous activity before sensory stimuli was recorded over a 100-s window. A solenoid-gated tube was positioned 2 cm above and 1 cm to the side of the nose to deflect the large facial whiskers. Stimulus position and air puff intensity was calibrated and held constant over days. Responses to either a vertical air puff [500-ms duration, 6 psi (41.37 kPa)] or blank (solenoid click) delivered by Arduino every 20 s (0.05 Hz) to the right-side whiskers during each session were obtained. Air puff and blank stimuli had an equal probability of occurring and were randomly interleaved. We collected another 100 s of spontaneous activity following stimulation. Following the imaging sessions, mice were promptly returned to their home-cage training environment to minimize disruption to their daily routine and ensure the stability of their behavioral training regimen. Behavioral data from one mouse in the SAT SST-Cre × Ai32 dataset and three mice in the SAT SST-Flp × Calb2-Cre dataset were corrupted and unable to be analyzed. Animal participation in training could be deduced through tracking water consumption each day.

After animal training and when all imaging sessions were completed, the head bracket and window were removed, and the imaging site was marked by marking the site with a glass micropipette containing methylene blue dye. Brains were fixed in 4% paraformaldehyde and sectioned either coronally or flattened and cut tangentially to confirm the imaging site location.

### 2P recording analyses

An imaging file containing all imaging sessions (~96,000 frames) was aligned and segmented with Suite2P (*37*). The output from Suite2P included all possible segments. Regions of interest (ROIs) were then manually selected from all segments based on morphology and fluorescence traces calculated by Suite2P. Individual ROIs (neurons) were tracked across each imaging day, and neurons that could not be tracked across all days were discarded from the analysis.

Image movement was assessed by calculating shifts in aligned pixels across frames, extracted from Suite2P. We established that any frame that shifted more than 20 pixels in either the *x* or *y* direction within the larger FOV was considered a shifted frame. One or two continuously shifted frames were interpolated with the average value of the previous and the next unshifted frames (both fluorescence signal and pixel shift). When >3 consecutive frames were shifted within a single trial, the trial was then removed.

Raw fluorescence was extracted for each segmented ROI, and fluorescence signals were neuropil corrected (*F*_corrected_ = FROI − 0.7 × *F*_neuropil_) to remove a contribution from SST neurons in other layers (*38*). For quantification of the evoked response peak, baseline fluorescence (*F*_0_) was calculated by averaging the neuropil-corrected signal (*F*_corrected_) within a 1-s time window preceding the stimulus onset of individual trials. The stimulus-evoked change in fluorescence relative to baseline, Δ*F*/*F*_0_, was computed for each trial using *F*_corrected_ − *F*_0_/*F*_0_. Individual neuropil-corrected ROIs were designated as neurons. All stimulus trials, irrespective of the amplitude of the response, were used to calculate the mean peak response for a given neuron.

The daily stimulus-evoked activity of each neuron was calculated by averaging the cell response across all stimulus trials within each imaging day. The peak response from ACC4 to ACC6 was used to normalize responses from the SAT period since neural activity during the first three imaging days showed greater variability than subsequent days of imaging in the pretraining period.

### Classification of calretinin (Calb2^+^) neurons

To identify Calb2 neurons from in vivo imaging FOVs in SST-Flp × Calb2-Cre mice, an intensity matrix for each image plane was initially extracted from MES and subsequently transformed into a two-channel image featuring green (GCaMP6f) and red (mCherry) channels. The cell bodies of SST neurons were manually traced on the basis of GCaMP6f expression using ImageJ on both the initial (ACC1) imaging day within each imaging plane. Following this, the average pixel intensity of each corresponding region of interest in the mCherry channel was calculated. A cell was categorized as Calb2-positive if its average pixel intensity exceeded 200 arbitrary units on the initial imaging day (fig. S13).

### SST activity feature extraction

Activity features of neurons were extracted from GCaMP6f fluorescence signals from 10-min-long sessions, as described above, using only ACC4 to ACC6 imaging sessions. We only calculated first-order features, which are direct measurements of certain metrics from the fluorescence signal. Higher-order features, such as ratios or summations of different metrics, are not included in this study. As illustrated in fig. S19, starting from the raw neuropil-corrected signal, each session recording was divided into two distinct periods: trial periods and spontaneous activity blocks. Trial periods are time windows from −3 s to +5 s relative to each stimulus/blank trial onset. The spontaneous blocks comprise the remaining portion of the fluorescence signal. All features are generally divided into three main categories: response probability features, in-trial activity measurement features, and spontaneous activity measurement features.

Response probability features and in-trial activity measurement features are calculated from each single trial period and then averaged across each single day as the final feature vector value for each neuron on each day. Spontaneous activity features are calculated from each single block or each single detected event and then averaged across each single day as the final feature vector value for each neuron on each day. As described before, for trial period signals, the Δ*F*/*F*_0_ signal is calculated using a 1-s time window preceding trial onset as baseline fluorescence (*F*_0_). For spontaneous blocks, the Δ*F*/*F*_0_ signal is calculated using a 1-min sliding window with a 20th percentile filter as baseline fluorescence (*F*_0_).

Features were calculated using various parameters, including different timing periods, trial types, spontaneous block types, and thresholding methods. The full-feature space encompasses all possible combinations of these parameter setups. Thresholds for response probability features (σ_1_) and spontaneous event detection (σ_2_) were calculated differently. Specifically, σ_1_ (for responsive trial detection) was determined from the standard deviation of concatenated baseline periods (1 s before trial onset) across all trials’ Δ*F*/*F*_0_ signals. In contrast, σ_2_ (for spontaneous activity detection) was the overall standard deviation of the entire session’s Δ*F*/*F*_0_ signal trace. In feature extraction, thresholds were set as different multiples of σ_1_ and σ_2_, ranging from 1 to 10 SDs. For response probability features and in-trial activity measurement features, different options were considered, including the trial types and different in-trial periods, such as the pretrial period (−2 to 0 s relative to trial onset) of all stimulus trials or the posttrial period (2 to 4 s relative to trial onset) of all blank trials. For response probability features, due to broad variation in the distribution of peak response amplitudes and background fluctuations in fluorescence across individual neurons in our dataset, different thresholds including 1σ_1_, 2σ_1_, 3σ_1_, 5σ_1_, and 10σ_1_ were adopted to detect responsive trials. For in-trial measurement features, different metrics used included peak, peak latency, center of mass, and area under the curve. For the spontaneous activity period, peaks were detected using SciPy’s find_peaks function (version 1.11.1) under different multiples of σ_2_. Events were detected when their prominence exceeded a threshold set as a multiple of σ_2_. Spontaneous activity features were also calculated across different time periods, including the initial block (the 100-s window before the task started), intertrial blocks (starting from 5 s after the previous trial ends to 3 s before the next trial starts), and final blocks (the 100-s window after the task ends). The spontaneous activity metrics include the peak amplitude of each detected event, the number of events, and the area under the curve for each spontaneous activity block. Further details of the extracted features can be found in tables S1 to S3. After feature extraction, we sorted the features based on their predictive ability to separate SST-Calb2 cells from SST-O cells, using unpaired *t* tests at the level of each individual feature. The ranking of all features and example showcases can be found in fig. S20.

### Uniform Manifold Approximation and Projection clustering and clustering-informed heuristic classification

Fixed-parameter classifiers can fail to perform robustly across varied experimental conditions. Differences in mouse lines, calcium imaging environments, and equipment can significantly alter fluorescence distributions, introducing hard-to-quantify shifts in the data. As a result, these static classifiers are prone to overfitting, particularly with small datasets, and often fail to capture the underlying structure of heterogeneous datasets, contributing to nonreproducibility across studies. Thus, we chose to use clustering-informed heuristic classification to investigate the intrinsic structure of SST activity profiles present in both transgenic and virally expressed GCaMP6f signals from pretraining data (ACC4 to ACC6).

On the basis of the extracted features, Uniform Manifold Approximation and Projection (UMAP; version 0.5.3) dimensionality reduction was applied to the top 30 predictive features, irrespective of their categories (fig. S20). Features extracted from single units (single trials or single spontaneous blocks) were averaged across daily recordings from ACC4 to ACC6 and then *z*-score normalized to ensure equal feature contributions to the final clustering. The dimension reduction result embeddings were further clustered using different clustering methods from the SciPy package, including DBSCAN (which requires no prespecified cluster number), *k*-means, and spectral clustering (which require prespecified cluster numbers). We applied grid search across all hyperparameters mentioned in the previous pipeline, including n_neighbors and min_dist in UMAP, maximum distance (eps) in DBSCAN, and cluster numbers in KMeans and spectral clustering. Representative clustering results were selected on the basis of their silhouette scores and labeling consistency with the transcriptomic hypothesis. Justification for choosing three clusters as the representative clustering solution is provided in fig. S21, where a much larger grid search space was applied, testing ~5000 different UMAP embedding and clustering labeling combinations. Results indicated three as a robust choice. To reproduce our results or further explore the heuristic clustering method, please refer to the available code on GitHub: https://github.com/barthlab/Long-lasting-subtype-specific-regulation-of-somatostatin-interneurons-during-sensory-learning.

### Evaluation of classifier accuracy

Our clustering-based analysis uses an unsupervised, parameter-free heuristic rather than a supervised classification model and thus does not involve conventional training-testing comparisons. Given the limited number of cells in our datasets (84 cells for the labeled SAT group and 98 cells for the unlabeled SAT group), any cross-validation would risk overfitting. On the basis of the representative clustering labeling presented in Fig. 5, the true positive count is 18, false positive is 11, true negative is 38, and false negative is 17, resulting in an F_1_ score of 0.562 and 67% accuracy (for SST-Calb2, 51% recall and 62% precision; for SST-O, 77% recall and 69% precision. The high true-negative rate suggests effective identification of Calb2-negative SST cells. However, the low true-positive rate—likely due to SST-Calb2 cell heterogeneity (see Fig. 5, clustering)—indicates the potential for further subdivision within the SST-Calb2 population.

### Tissue collection for anatomical analyses

To control for the transduction time of PSD95.FingR, mice were euthanized 14 days after viral injections at midday regardless of experimental condition. Mice were deeply anesthetized with a near lethal dose of isoflurane and transcardially perfused with 20 ml of 1× phosphate-buffered saline (PBS), followed by 20 ml of 4% paraformaldehyde in 1× PBS. Brains were carefully removed and postfixed in 4% paraformaldehyde overnight, followed by transfer to 30% sucrose in 1× PBS. Approximately 3 days following sucrose immersion, ~50-μm-thick free-floating sections were acquired using a freezing microtome (Leica Biosystems) and stored in PBS. Sections typically underwent immunohistochemical staining within 2 days of slicing.

### Immunohistochemistry

Before staining, four alternating sections containing posterior barrel cortex were washed in 1× PBS (Boston BioProducts Inc.) for 5 min over 5 cycles. Sections were shaken in a blocking solution containing 1× PBS, 10% goat serum (Sigma-Aldrich) and 0.3% Triton X-100 (Sigma-Aldrich) in MilliQ water for 2 hours. After blocking, a 1:500 dilution of rabbit α-calretinin primary antibody (Swant, #CR7697) was mixed with the block solution containing 5% goat serum instead of 10%. Sections were covered and placed on a rocker at 4°C overnight (20 to ~24 hours). After the primary, sections were washed in 1× PBS for 5 min over 5 cycles. Last, sections were shaken in a 1:500 dilution of far-red secondary antibody (CF640Rα rabbit) in 1× PBS for 2 hours, followed by a final step of 1× PBS washes. Sections were immediately mounted in antifade mounting medium containing 4ont-diamidino-2-phenylindole (DAPI) (VECTASHIELD) on Diamond White Glass charged slides (Globe Scientific).

### Confocal image acquisition

FOVs were collected using an LSM 880 Axio Observer microscope (Carl Ziess). Using a 10× objective, FOVs were coarsely targeted over S1BF using barrels resolved by DAPI staining as a guide. Precise targeting of L2/3 FOVs under the 63× oil immersion objective lens (Plan-Apochromat, NA 1.40, oil) was done using the granular layer (start of L4) demarcated by DAPI staining as the lower bound of and the steep drop-off in SST dendritic arborizations as the upper bound of L2/3 (bottom of L1). L4 was targeted using an increase in DAPI signal as a marker and generally 400 to 500 μm from the pial surface, while L5 was characterized by an increase in density of SST somas. Volumetric stacks using the 63× oil immersion objective lens, set at a 0.9 zoom factor and 1.0 Airy disk unit, were used to collect ~100 images with 1024 pixel–by–1024 pixel resolution with a *z*-step size of .3um. This resulted in a 149.54 μm–by–149.54 μm–by–29.7 μm image stack with the voxel dimensions of 0.146 μm–by–0.146 μm–by–0.3 μm. PSD95.FingR-citrine fluorescence was collected for later surface reconstruction analysis (excitation, 514 nm; emission, 517 to 561 nm). For experiments where PSD95.FingR puncta were registered with Calb2 identity, the far-red channel (excitation, 640 nm; emission, 641 to 695 nm) was also captured. Laser power (514 nm) was adjusted (generally between 4 and 6%) for each FOV to prevent over- or undersaturation of punctate PSD95.FingR pixel intensities. The gain was set to be between 700 and 720 arbitrary units across all animals.

### Digital reconstruction of fluorescent signal

Volumetric stacks containing PSD95.FingR-citrine–labeled excitatory synapses were analyzed using the image analysis software Imaris (version 8.4.1, Bitplane). The citrine channel was adjusted such that background signal was subtracted to resolve PSD95.FingR puncta. This fluorescent channel was then digitally reconstructed using the Imaris watershedding algorithm (Surfaces macro; image segmentation). The watershed threshold for PSD95 puncta signal (without smoothing; expected size, 0.5 μm) was adjusted to maximize coverage of fluorescence signal while minimizing reconstructing signal contained in the background. Fused objects (adjacent overlapping signal) were split using the quality filter feature built into the surface macro (0.45 μm). Surfaces were then filtered by voxel size (>3 voxels) to minimize noise captured in surface reconstructions. In cases where all PSD95 puncta from an FOV were included in the analysis, reconstructions resulting from somatic and nuclear citrine fluorescent signal were removed by filtering surfaces of <0.15 μm from reconstructed somas (watershed threshold: smoothing, 0.263 arbitrary units; no quality filter; voxel size, adjusted to include fully reconstructed somas).

### Confocal image analysis

Following digital reconstructions of fluorescent signal, characteristics of PSD95 puncta surfaces could be quantified and compared across conditions. Volumes of each reconstructed surface were obtained to estimate the size of putative excitatory synapses. These puncta were averaged together to obtain a mean size of excitatory puncta for a given animal or cell type. For large-scale FOV analyses (fig. S9), unlabeled puncta obtained from each animal were randomized and balanced across conditions.

To quantify the difference between levels of excitation in SST-Calb2 versus SST-O neurons, we registered puncta as belonging to somas that were positive or negative for immunohistochemically labeled calretinin (protein product of the Calb2 gene). Since SST neurons are relatively sparse, PSD95.FingR puncta on individual dendrites that emanate from an individual soma could be resolved. Following semiautomated reconstruction of the entire field of PSD95 surfaces, ~200 puncta were manually selected along a stretch of dendrites emanating from a given soma of each cell type. Experimenters were blind to cell type identity and condition during manual selection and later categorized puncta as belonging to Calb2 positive or negative neurons based on somatic calretinin expression. Both groups of SST neurons were captured in the same FOV, and the image acquisition and reconstruction settings were held constant, which enabled within-sample comparison between groups.

### Slice preparation

Animals were anesthetized with isoflurane briefly and decapitated between 11 a.m. and 2 p.m. Off-coronal slices with a thickness of 350 μm (one cut, 45° rostrolateral) were prepared in ice-cold artificial cerebrospinal fluid (ACSF) composed of 119 mM NaCl, 2.5 mM KCl, 1 mM NaH_2_PO_4_, 26.2 mM NaHCO_3_, 11 mM glucose, 1.3 mM MgSO_4_, and 2.5 mM CaCl_2_ equilibrated with 95%/5% O_2_/CO_2_. Tissues were recovered at room temperature in cutting ACSF for 45 min to 1 hour before recording.

### General electrophysiology

Recordings were performed in cutting ACSF in the presence of synaptic blockers (10 μM 2,3-dioxo-6-nitro-7-sulfamoylbenzo[*f*]quinoxaline, 50 μM d-aminophosphovalerate, and 50 μM pertussis toxin). Cortical SST neurons were targeted using an Olympus light microscope (BX51WI) and borosilicate glass electrodes (4- to 9-megohm pipette resistance) filled with internal solution composed of 125 mM potassium gluconate, 10 mM Hepes, 2 mM KCl, 0.5 mM EGTA, 4 mM Mg–adenosine 5′-triphosphate, 0.3 mM Na–guanosine 5′-triphosphate, and trace amounts of Alexa Fluor 568 (pH 7.25 to 7.30, 290 mOsm) for morphological confirmation of cell identity. Electrophysiological data were acquired using Multiclamp 700B amplifier (Axon Instruments) and digitized with a National Instruments acquisition interface (National Instruments). Multiclamp and IgorPro 6.0 software (WaveMetrics) with 3-kHz filtering and 10-kHz digitization were used to collect data.

ChR2-expressing L2/3 SST neurons were targeted using reporter fluorescence. After breaking into the cells, SST neurons were voltage clamped at −70 mV for 3 to 5 min until the baseline membrane potential stabilized. Action potentials were evoked with depolarizing current steps (25, 50, 100, 150, 200, 250, and 300 pA for 500-ms duration, three sweeps each at 0.1 Hz) in current clamp at −60 mV. Cell identity was confirmed by enhanced yellow fluorescent protein fluorescence, low-threshold spiking firing phenotype, and light-evoked spikes. Only SST neurons with membrane potential ≤−40 mV and a stable membrane potential baseline were included in our analysis. To calculate spiking frequency, depolarizations that exceed 0 mV were counted as action potentials. The number of evoked action potentials was calculated by averaging three sweeps for each current injection amplitude. Resting membrane potential was obtained once cells had stabilized. Input resistance (in megohms) was calculated for each trial and averaged across all excitability experiment trials. Rheobase (in picoamperes) was determined as the minimum current required to elicit a single spike.
