# Supplementary material for: Long-lasting, subtype-specific regulation of somatostatin interneurons during sensory learning
Source: Sci Adv. 2025 Aug 15;11(33):eadt8956. doi: 10.1126/sciadv.adt8956 (PMC12356249; doi:10.1126/sciadv.adt8956)
Supplement: Supplementary file 1 — Supplementary Materials and Methods Figs. S1 to S22 Tables S1 to S3 [file sciadv.adt8956_sm.pdf]

Science Advances
AAAS

Supplementary Materials for

Long-lasting, subtype-specific regulation of somatostatin interneurons during sensory learning

Matthew B. Mosso *et al.*

Corresponding author: Alison L. Barth, barth@cmu.edu

Sci. Adv. **11**, eadt8956 (2025)
DOI: 10.1126/sciadv.adt8956

This PDF file includes:

Supplementary Materials and Methods
Figs. S1 to S22
Tables S1 to S3

Materials and Methods

Animals

For GCaMP6f imaging in somatostatin neurons, we crossed Sst-IRES-Cre mice (Jackson #013044) to Ai148(TIT2L-GC6f-ICL-tTA2)-D mice (Jackson #030328). For GCaMP6f imaging in calretinin-expressing somatostatin neurons, we crossed Sst-IRES-Flp mice (Jackson #028579) to Cr-IRES-Cre (Calb2-IRES-Cre) mice (Jackson #010774). Juvenile to adult transgenic mice (1.5-6 mos of age) were used for cranial window surgery and virus injection. They recovered for 1-3 weeks before commencing 2P *in vivo* imaging. Sst-IRES-Cre mice (Jackson #013044) were used for fixed tissue analysis. Male and female mice were used for all experiments and approximately balanced across control and experimental datasets. All procedures for animal experiments were performed following protocols (PROTO201600045) approved by the Institutional Animal Care & Use Committee at Carnegie Mellon University and were carried out in accordance with US National Institutes of Health guidelines.

Cranial window surgery

Surgery was done under isoflurane anesthesia (4% for induction, 1.5-2% for maintenance). Mice were put on a heat pad with a temperature control system (FHC 40-90-8D) to maintain body temperature. Eyes were covered with Puralube Vet Ointment to prevent drying. Fur was removed with Nair, and the skin was cleaned with povidone and then incised expose the skull. The skull was scraped with a dental blade (Salvin 6900) to remove the periosteum and abraid the surface for headpost attachment. On the left hemisphere, S1 coordinates (3.5 mm lateral, 1 mm posterior to bregma) and a 3 mm diameter circle centered at the coordinates were marked with a pen. A thin layer of tissue adhesive (3M VetBond) was applied to the skull, then a custom-made headpost was attached to the right hemisphere with cyanoacrylate glue and dental cement (Lang Dental, 1223PNK). With a dental drill (Dentsply, 780044), the skull was thinned along the 3 mm diameter circle. Thinned skull was removed by lifting a spot of the thinned region with forceps. Minor bleeding was stopped with saline-soaked gelfoam (Pfizer, 00009032301), and a glass window comprised of a 3 mm diameter glass (Warner Instruments, 64-0726) attached to a 4 mm diameter glass (Warner Instruments, 64-0724) by UV adhesive (Norland, 717106) was applied over the craniotomy. The window was sealed with 3M Vetbond and then cyanoacrylate glue. All exposed skull area except the window was covered with dental cement. A well surrounding the window was built with dental cement for microscopy using a water immersion lens. At the end of the surgery, ketoprofen (3 mg/kg) was injected subcutaneously, and the mouse was allowed to recover in a heated cage. Mice were given 1-3 weeks of recovery before imaging commenced.

Stereotaxic injections

Male and female mice expressing Cre recombinase under the somatostatin promoter aged P50-P100 were induced into anesthesia with 4% isoflurane and administered a maintenance dose of ~1.5% isoflurane throughout the duration of surgery. Using a dental drill, a burr hole was drilled preserving the final layer of skull to minimize damage to the cortex. Saline was washed over the skull to soften the injection zone prior to insertion of the glass pipette. Mice were injected with ~80nL of AAV-PHP.eB-ZFN-hSyn-DIO-PSD95.FingR-Citrine-reg.WPRE into S1 (-3.5mm lateral, -1.25mm posterior relative to lambda) using a nanoject (Drummond Scientific). Post injection, sutures were used to close the scalp lesion. Following surgery, mice were single

housed to reduce possible confounds of different socialization levels among mice compared across trained or naïve conditions.

For chemogenetic experiments designed to monitor changes in PSD95 puncta after suppressing activity, we coinjected pAAV8-hSyn-DIO-hM4Di-mCherry (Adgene #44362) with AAV-PHP.eB-ZFN-hSyn-DIO-PSD95.FingR-Citrine-reg.WPRE into S1BF using the stereotaxic injection procedures outlined above. Aliquots of Clozapine-N-Oxide (CNO; ApexBio) made up in DMSO (0.01mg/ul) were placed in the drinking water to approximate the dosage of 1mg/kg per day based on animal initial weight and estimated water consumption of 2 ml per day. To administer CNO, mice were placed in the training cage without any airpuff cue and were freely able to collect water containing CNO for five days. Like the acclimation period in other SAT training experiments, trials dispensed water at 80% probability.

For GCaMP6f imaging and calretinin labelling in SST neurons using viral methods, we injected AAV1-Ef1a-fDIO-GCaMP6f (Addgene #128315) mixed at a 1:1 ratio with AAV8-Ef1a-Con/Fon-mCherry (Addgene #137132) or pAAV8-hsyn-DIO-mCherry (Addgene #50459) in Sst-IRES-Flp x Cr-IRES-Cre (Calb2-IRES-Cre) mice using a Nanoject II (Drummond Scientific) directly before application of the cranial window implant. After craniotomy, a ~0.6 μL of virus (~1.84×10^{13} vg/mL) was injected into the barrel cortex (three sites across the cranial window, 0.3 mm below the pial surface). The virus expression period lasted 26-42 days prior to the commencement of imaging.

Sensory association training (SAT)

Mice were trained to associate a multiwhisker stimulus with a delayed water reward in an automated training cage (*15*, *35*). Mice were single-housed in a home cage connected to a freely-accessible chamber with a water port and an airpuff delivery tube. Animals were not water deprived. During the cage acclimation period, animals could freely approach the lickport and initiate a trial by breaking an infrared beam. During the acclimation period, nosepokes initiated a random delay period lasting 1.2-1.8s, followed by delivery of a water droplet (~10 ul) dispensed at 80% probability (i.e. 20% of nosepokes did not result in water delivery). During SAT, 80% of beambreaks were followed by a random delay 0.2-0.8s and then a gentle airpuff (6 psi, 500 ms duration) delivered to the right-side whiskers, followed 500 ms delay and then water delivery (fig. S1). This training paradigm has been described in detail elsewhere (*15*, *20*, *35*). Anticipatory licking frequency was assessed during the 300 ms period immediately prior to water delivery. The sensory cue was fully predictive; i.e. all airpuff stimuli were followed by water. During SAT, 80% of trials consistent of the predictive airpuff followed by the water reward. The remaining 20% of trials had no stimulus and no water reward (blank trials). There was a 2 s timeout between trials, where nosepokes would not trigger water delivery. Animals were freely-moving and lived in the training cages except during daily imaging periods. Mice typically carried out ~500 trials per day.

During pseudotraining, the airpuff stimulus randomly preceded either water or blank trials, so that airpuff had no predictive value for water delivery. During the acclimation period, water was delivered with a 50% probability to match the probability used for pseudotraining. During pseudotraining, airpuff was delivered in 80% of the trials but water followed the stimulus for only half the trials while the other half of stimulus trials were followed by no water delivery (fig. S3; (*15*). To further decouple the stimulus from the reward, water was delivered without a

preceding airpuff for half of the remaining non-stimulus trials. Therefore, the airpuff stimulus and water reward were entirely decoupled during pseudotraining. Animal performance was calculated as described for SAT.

Both SAT and pseudotrained animals undergoing 2P imaging experienced 6 days of acclimation and then 10 days of training. For each animal, total number of trials (water + blank trials) and anticipatory lick frequencies (licks occurring in a 300 ms window right before water delivery; see (*35*)) were calculated for every 4-hour bin using a custom MATLAB code. Any 4-hour bin with fewer than 10 trials was removed from the averaged data, since lick frequency on blank trials could not be accurately assessed from 1-2 trials. Performance was calculated by taking the difference between anticipatory licking frequencies (Hz) during stimulus versus blank trials ($licking_{stimulus}$-$licking_{blank}$). Absolute differences in calculated lick frequency between stimulus and blank trials for the last 20% of trials on a given day were compared using a Wilcoxin signed-rank test.

2P *in vivo* imaging

All training sessions were conducted within the automated homecage training system, ensuring a consistent and controlled environment for behavioral learning tasks. For 2P imaging experiments, mice were removed from their homecage environment for brief periods of 1 hour per day, typically around noon. No lickport was present during training, and the number of stimulus trials was kept to a minimum (~15) to prevent extinction. No difference in performance after imaging sessions was observed, indicating that this brief exposure did not alter the learned association (*36*). Mice were removed from the training cage around noon each day, briefly anaesthetized with volatile isofluorane (4% for ~20 s) to headfix the animal under the microscope, and then allowed to recover for 3-5 minutes before imaging. Animals were awake and ambulatory on the wheel before imaging began. Imaging was carried out with 2P microscope (Femto2D Galvo), equipped with a Mai Tai laser MTEV HP 1040S (Spectra-Physics), a 4x air objective lens (Olympus UPLFLN 4X NA 0.13), and a 40x water objective lens (Olympus LUMPLFLN 40XW NA 0.8). Images were acquired with MES software v.6.1.4306 (Femtonics).

Blood vessel morphology in 4x brightfield was used to find the same imaging field of view (FOV) as the previous session. The pial surface (z=0) was defined as the plane right below the dura mater which looks like a textured membrane in 40x brightfield. In 40x 2P mode, the x, y, z positions of the neurons were aligned to match the previous session image. A 950 nm excitation was used to image GCaMP6f signals, and emission fluorescence was detected with photomultiplier tube (PMT; Hamamatsu H11706P-40). Laser power and PMT voltage were kept constant for each animal across its imaging sessions. Images were acquired at 5.11 Hz with ~270 µm x 300 µm FOV and 0.7 µm/pixel resolution. Imaging depth was ~200 µm below pia (L2/3), and 1-2 FOVs were imaged per mouse.

For each day, approximately 3-5 minutes after head fixation, 1-2, 10-minute imaging sessions were carried out, with a 1-minute break in between. At the beginning of each imaging session, spontaneous activity prior to sensory stimuli was recorded over a 100s window. A solenoid-gated tube was positioned 2 cm above and 1 cm to the side of the nose, in order to deflect the large facial whiskers. Stimulus position and airpuff intensity was calibrated and held constant over days. Responses to either a vertical airpuff (500 ms duration, 6 psi) or blank (solenoid click) delivered by Arduino every 20 s (0.05 Hz) to the right-side whiskers during each session

were obtained. Airpuff and blank stimuli had an equal probability of occurring and were randomly interleaved. We collected another 100s of spontaneous activity following stimulation. Following the imaging sessions, mice were promptly returned to their homecage training environment to minimize disruption to their daily routine and ensure the stability of their behavioral training regimen. Behavioral data from one mouse in the SAT SST-Cre x Ai32 dataset and 3 mice in the SAT SST-Flp x Calb2-Cre dataset were corrupted and unable to be analyzed. Animal participation in training could be deduced through tracking water consumption each day.

After animal training and when all imaging sessions were completed, the head bracket and window were removed, and the imaging site was marked by marking the site with a glass micropipette containing methylene blue dye. Brains were fixed in 4% paraformaldehyde and sectioned either coronally or flattened and cut tangentially to confirm the imaging site location.

2P recording analyses

An imaging file containing all imaging sessions (~96000 frames) was aligned and segmented with Suite2P (*37*). The output from Suite2P included all possible segments. ROIs were then manually selected from all segments based on morphology and fluorescence traces calculated by Suite2P. Individual regions of interest (ROIs) (neurons) were tracked across each imaging day, and neurons that could not be tracked across all days were discarded from the analysis.

Image movement was assessed by calculating shifts in aligned pixels across frames, extracted from Suite2P. We established that any frame that shifted more than 20 pixels in either the X or Y direction within the larger FOV was considered a shifted frame. One or two continuously shifted frames were interpolated with the average value of the previous and the next unshifted frames (both fluorescence signal and pixel shift). When >3 consecutive frames were shifted within a single trial, the trial was then removed.

Raw fluorescence was extracted for each segmented ROI, and fluorescence signals were neuropil-corrected ($F_{corrected}$ =FROI – 0.7*$F_{neuropil}$) to remove a contribution from SST neurons in other layers (*38*). For quantification of the evoked response peak, baseline fluorescence (F_0) was calculated by averaging the neuropil-corrected signal ($F_{corrected}$) within a 1s time window preceding the stimulus onset of individual trials. The stimulus-evoked change in fluorescence relative to baseline, $\Delta F/F_0$, was computed for each trial using $F_{corrected}$ - F_0/F_0. Individual neuropil-corrected ROIs were designated as neurons. All stimulus trials, irrespective of the amplitude of the response, were used to calculate the mean peak response for a given neuron.

The daily stimulus-evoked activity of each neuron was calculated by averaging the cell response across all stimulus trials within each imaging day. The peak response from ACC4-6 was used to normalize responses from the SAT period since neural activity during the first three imaging days showed greater variability than subsequent days of imaging in the pretraining period.

Classification of calretinin (Calb2+) neurons

To identify Calb2 neurons from *in vivo* imaging FOVs in SST-Flp x Calb2-Cre mice, an intensity matrix for each image plane was initially extracted from MES and subsequently transformed into

a two-channel image featuring green (GCaMP6f) and red (mCherry) channels. The cell bodies of SST neurons were manually traced based on GCaMP6f expression using ImageJ on both the initial (ACC1) imaging day within each imaging plane. Following this, the average pixel intensity of each corresponding region of interest in the mCherry channel was calculated. A cell was categorized as Calb2 positive if its average pixel intensity exceeded 200 A.U. on the initial imaging day (fig. S13).

SST activity feature extraction

Activity features of neurons were extracted from GCaMP6f fluorescence signals from 10-minute-long sessions, as described above, using only ACC4-6 imaging sessions. We only calculated first-order features, which are direct measurements of certain metrics from the fluorescence signal. Higher-order features, such as ratios or summations of different metrics, are not included in this study. As illustrated in fig. S19, starting from the raw neuropil-corrected signal, each session recording was divided into two distinct periods: trial periods and spontaneous activity blocks. Trial periods are time windows from -3s to +5s relative to each stimulus/blank trial onset. The spontaneous blocks comprise the remaining portion of the fluorescence signal. All features are generally divided into three main categories: response probability features, in-trial activity measurement features, and spontaneous activity measurement features.

Response probability features and in-trial activity measurement features are calculated from each single trial period and then averaged across each single day as the final feature vector value for each neuron on each day. Spontaneous activity features are calculated from each single block or each single detected event and then averaged across each single day as the final feature vector value for each neuron on each day. As described before, for trial period signals, the $\Delta F/F_0$ signal is calculated using a 1s time window preceding trial onset as baseline fluorescence (F_0). For spontaneous blocks, the $\Delta F/F_0$ signal is calculated using a 1-minute sliding window with a 20th percentile filter as baseline fluorescence (F_0).

Features were calculated using various parameters, including different timing periods, trial types, spontaneous block types, and thresholding methods. The full feature space encompasses all possible combinations of these parameter setups. Thresholds for response probability features (σ_1) and spontaneous event detection (σ_2) were calculated differently. Specifically, σ_1 (for responsive trial detection) was determined from the standard deviation of concatenated baseline periods (1s before trial onset) across all trials' $\Delta F/F_0$ signals. In contrast, σ_2 (for spontaneous activity detection) was the overall standard deviation of the entire session's $\Delta F/F_0$ signal trace. In feature extraction, thresholds were set as different multiples of σ_1 and σ_2, ranging from 1 to 10 standard deviations. For response probability features and in-trial activity measurement features, different options were considered, including the trial types and different in-trial periods, such as the pre-trial period (-2~0s relative to trial onset) of all stimulus trials or the post-trial period (2~4s relative to trial onset) of all blank trials. For response probability features, due to broad variation in the distribution of peak response amplitudes and background fluctuations in fluorescence across individual neurons in our dataset, different thresholds including $1\sigma_1$, $2\sigma_1$, $3\sigma_1$, $5\sigma_1$, and $10\sigma_1$ were adopted to detect responsive trials. For in-trial measurement features, different metrics used included peak, peak latency, center of mass, and area under the curve. For the spontaneous activity period, peaks were detected using SciPy's find_peaks function (version 1.11.1) under different multiples of σ_2. Events were detected when their prominence exceeded a

threshold set as a multiple of σ_2. Spontaneous activity features were also calculated across different time periods, including the initial block (the 100s window before the task started), inter-trial blocks (starting from 5s after the previous trial ends to 3s before the next trial starts), and final blocks (the 100s window after the task ends). The spontaneous activity metrics include the peak amplitude of each detected event, the number of events, and the area under the curve for each spontaneous activity block. Further details of the extracted features can be found in Tables 1–3. After feature extraction, we sorted the features based on their predictive ability to separate SST-Calb2 cells from SST-O cells, using unpaired t-tests at the level of each individual feature. The ranking of all features and example showcases can be found in Fig. S20.

UMAP clustering and clustering-informed heuristic classification

Fixed-parameter classifiers can fail to perform robustly across varied experimental conditions. Differences in mouse lines, calcium imaging environments, and equipment can significantly alter fluorescence distributions, introducing hard-to-quantify shifts in the data. As a result, these static classifiers are prone to overfitting, particularly with small datasets, and often fail to capture the underlying structure of heterogeneous datasets, contributing to non-reproducibility across studies. Thus, we chose to use clustering-informed heuristic classification to investigate the intrinsic structure of SST activity profiles present in both transgenic and virally expressed GCaMP6f signals from pretraining data (ACC4-6).

Based on the extracted features, Uniform Manifold Approximation and Projection (UMAP, version 0.5.3) dimensionality reduction was applied to the top 30 predictive features, irrespective of their categories (Fig. S20). Features extracted from single units (single trials or single spontaneous blocks) were averaged across daily recordings from ACC4-6 and then z-score normalized to ensure equal feature contributions to the final clustering. The dimension reduction result embeddings were further clustered using different clustering methods from the SciPy package, including DBSCAN (which requires no pre-specified cluster number), KMeans, and Spectral Clustering (which require pre-specified cluster numbers). We applied grid search across all hyperparameters mentioned in the previous pipeline, including n_neighbors and min_dist in UMAP, maximum distance (eps) in DBSCAN, and cluster numbers in KMeans and Spectral Clustering. Representative clustering results were selected based on their Silhouette scores and labeling consistency with the transcriptomic hypothesis. Justification for choosing three clusters as the representative clustering solution is provided in fig. S21, where a much larger grid search space was applied, testing ~5,000 different UMAP embedding and clustering labeling combinations. Results indicated three as a robust choice. To reproduce our results or further explore the heuristic clustering method, please refer to the available code on GitHub: https://github.com/barthlab/Long-lasting-subtype-specific-regulation-of-somatostatin-interneurons-during-sensory-learning

Evaluation of classifier accuracy

Our clustering-based analysis employs an unsupervised, parameter-free heuristic rather than a supervised classification model, and thus does not involve conventional training-testing comparisons. Given the limited number of cells in our datasets (84 cells for the labeled SAT group and 98 cells for the unlabeled SAT group), any cross-validation would risk overfitting. Based on the representative clustering labeling presented in Fig. 5, the true positive count is 18, false positive is 11, true negative is 38, and false negative is 17, resulting in an F1-score of 0.562

and 67% accuracy. (For SST-Calb2: 51% recall and 62% precision; for SST-O: 77% recall and 69% precision.) The high true negative rate suggests effective identification of Calb2-negative SST cells. However, the low true positive rate—likely due to SST-Calb2 cell heterogeneity (see Fig. 5 clustering)—indicates the potential for further subdivision within the SST-Calb2 population.

Tissue collection for anatomical analyses

To control for the transduction time of PSD95.FingR, mice were sacrificed 14 days after viral injections at midday regardless of experimental condition. Mice were deeply anesthetized with a near lethal dose of isoflurane and transcardially perfused with 20mL of 1x PBS followed by 20mL of 4% paraformaldehyde in 1x PBS. Brains were carefully removed and post-fixed in 4% PFA overnight followed by transfer to 30% sucrose in 1x PBS. Approximately three days following sucrose immersion ~50um thick free-floating sections were acquired using a freezing microtome (Leica Biosystems) and stored in PB. Sections typically underwent immunohistochemical staining within two days of slicing.

Immunohistochemistry

Prior to staining, four alternating sections containing posterior barrel cortex were washed in 1x PBS (Boston BioProducts Inc) for five minutes over five cycles. Sections were shaken in a blocking solution containing 1x PBS, 10% goat serum (Sigma-Aldrich) and .3% triton X (Sigma-Aldrich) in MilliQ water for 2 hours. After blocking, a 1:500 dilution of rabbit α calretinin primary antibody (Swant #CR7697) was mixed with the block solution containing 5% goat serum instead of 10%. Sections were covered and placed on a rocker at 4ºC overnight (20~24 hours). After the primary, sections were washed in 1x PBS for 5 minutes over five cycles. Finally, sections were shaken in a 1:500 dilution of Far-red secondary antibody (CF640R α Rabbit) in 1x PBS for 2 hours followed by a final step of 1x PBS washes. Sections were immediately mounted in antifade mounting media containing DAPI (Vectashield) on Diamond White Glass charged slides (Globe Scientific).

Confocal image acquisition

Fields of view (FOVs) were collected using an LSM 880 Axio Observer microscope (Carl Ziess). Using a 10x objective, FOVs were coarsely targeted over S1BF using barrels resolved by DAPI staining as a guide. Precise targeting of L2/3 FOVs under the 63x oil immersion objective lens (Plan-Apochromat, NA 1.40, oil) was done using the granular layer (start of L4) demarcated by DAPI staining as a lower bound of and the steep drop off in SST dendritic arborizations as an upper bound of L2/3 (bottom of L1). L4 was targeted by using an increase in DAPI signal as a marker and generally 400-500um from the pial surface while L5 was characterized by an increase in density of SST somas. Volumetric stacks using the 63x oil immersion objective lens set at a .9 zoom factor and 1.0 Airy disk unit was used to collect ~100 1024 x1024 pixel images with a z-step size of .3um. This resulted in a 149.54 x149.54 x 29.7um image stack with the voxel dimensions 0.146 x 0.146 x 0.3um. PSD95.FingR-Citrine fluorescence was collected for later surface reconstruction analysis (excitation 514nm, emission: 517-561nm). For experiments where PSD95.FingR puncta were registered with Calb2 identity, the far-red channel (excitation 640nm; Emission 641-695) was also captured. 514nm laser power was adjusted (generally

between 4-6%) for each FOV to prevent over-or under saturation of punctate PSD95.FingR pixel intensities. The gain was set to be between 700-720 arbitrary units across all animals.

Digital reconstruction of fluorescent signal

Volumetric stacks containing PSD95.FingR-Citrine labeled excitatory synapses were analyzed using the image analysis software Imaris (version 8.4.1; Bitplane). The citrine channel was adjusted such that background signal was subtracted to resolve PSD95.FingR puncta. This fluorescent channel was then digitally reconstructed using a the Imaris watershedding algorithm (Surfaces macro; image segmentation). The watershed threshold for PSD95 puncta signal (without smoothing; expected size 0.5um) was adjusted to maximize coverage of fluorescence signal while minimizing reconstructing signal contained in the background. Fused objects (adjacent overlapping signal) were split using the quality filter feature built into the surface macro (0.45um). Surfaces were then filtered by voxel size (>3 voxels) to minimize noise captured in surface reconstructions. In cases where all PSD95 puncta from a FOV were included in the analysis, reconstructions resulting from somatic and nuclear citrine fluorescent signal were removed by filtering surfaces <0.15um from reconstructed somas (watershed threshold: smoothing 0.263 arbitrary units; no quality filter; voxel size: adjusted to include fully reconstructed somas).

Confocal image analysis

Following digital reconstructions of fluorescent signal, characteristics of PSD95 puncta surfaces could be quantified and compared across conditions. Volumes of each reconstructed surface were obtained to estimate the size of putative excitatory synapses. These puncta were averaged together to obtain a mean size of excitatory puncta for a given animal or cell type. For large-scale FOV analyses (fig. S9), unlabeled puncta obtained from each animal were randomized and balanced across conditions.

To quantify the difference between levels of excitation in SST-Calb2 vs SST-O neurons, we registered puncta as belonging to somas that were positive or negative for immunohistochemically labeled calretinin (protein product of the Calb2 gene). Since SST neurons are relatively sparse, PSD95.FingR puncta on individual dendrites that emanate from an individual soma could be resolved. Following semi-automated reconstruction of the entire field of PSD95 surfaces, ~200 puncta were manually selected along a stretch of dendrites emanating from a given soma of each cell type. Experimenters were blind to cell-type identity and condition during manual selection and later categorized puncta as belonging to Calb2 positive or negative neurons based on somatic calretinin expression. Both groups of SST neurons were captured in the same FOV and the image acquisition and reconstruction settings were held constant which enabled within-sample comparison between groups.

Slice preparation

Animals were anesthetized with isoflurane briefly and decapitated between 11 am and 2 pm. 350μm thick off-coronal slices (one cut, 45° rostro-lateral) were prepared in ice-cold artificial cerebrospinal fluid (ACSF) composed of (mM): 119 NaCl, 2.5 KCl, 1 NaH2PO4, 26.2 NaHCO3, 11 glucose, 1.3 MgSO4, and 2.5 CaCl2 equilibrated with 95%/5% O2/CO2. Tissues were recovered at room temperature in cutting ACSF for 45 minutes to 1 hour before recording.

General Electrophysiology

Recordings were performed in cutting ACSF in the presence of synaptic blockers (10μM NBQX, 50μM D-APV and 50μM PTX). Cortical SST neurons were targeted using an Olympus light microscope (BX51WI) and borosilicate glass electrodes (4-9 MΩ pipette resistance) filled with internal solution composed of (in mM): 125 potassium gluconate, 10 HEPES, 2 KCl, 0.5 EGTA, 4 Mg-ATP, 0.3 Na-GTP, and trace amounts of AlexaFluor 568 (pH 7.25-7.30, 290 mOsm) for morphological confirmation of cell identity. Electrophysiological data were acquired using Multiclamp 700B amplifier (Axon Instruments) and digitized with a National Instruments acquisition interface (National Instruments). Multiclamp and IgorPro 6.0 software (Wavemetrics) with 3kHz filtering and 10kHz digitization were used to collect data.

ChR2-expressing L2/3 SST neurons were targeted using reporter fluorescence. After breaking into the cells, SST neurons were voltage-clamped at -70mV for 3-5 minutes until the baseline membrane potential stabilized. Action potentials were evoked with depolarizing current steps (25, 50, 100, 150, 200, 250, 300pA, for 500ms duration, 3 sweeps each at 0.1Hz) in current clamp at -60mV. Cell identity was confirmed by both eYFP fluorescence, low-threshold spiking firing phenotype and light-evoked spikes. Only SST neurons with membrane potential ≤-40mV and a stable membrane potential baseline were included in our analysis. To calculate spiking frequency, depolarizations that exceed 0 mV were counted as action potentials. The number of evoked action potentials was calculated by averaging 3 sweeps for each current injection amplitude. Resting membrane potential was obtained once cells had stabilized. Input resistance (MΩ) was calculated for each trial and averaged across all excitability experiment trials. Rheobase (pA) was determined as the minimum current required to elicit a single spike.

Fig. S1. Anticipatory licking reflects learning during sensory association training (SAT)

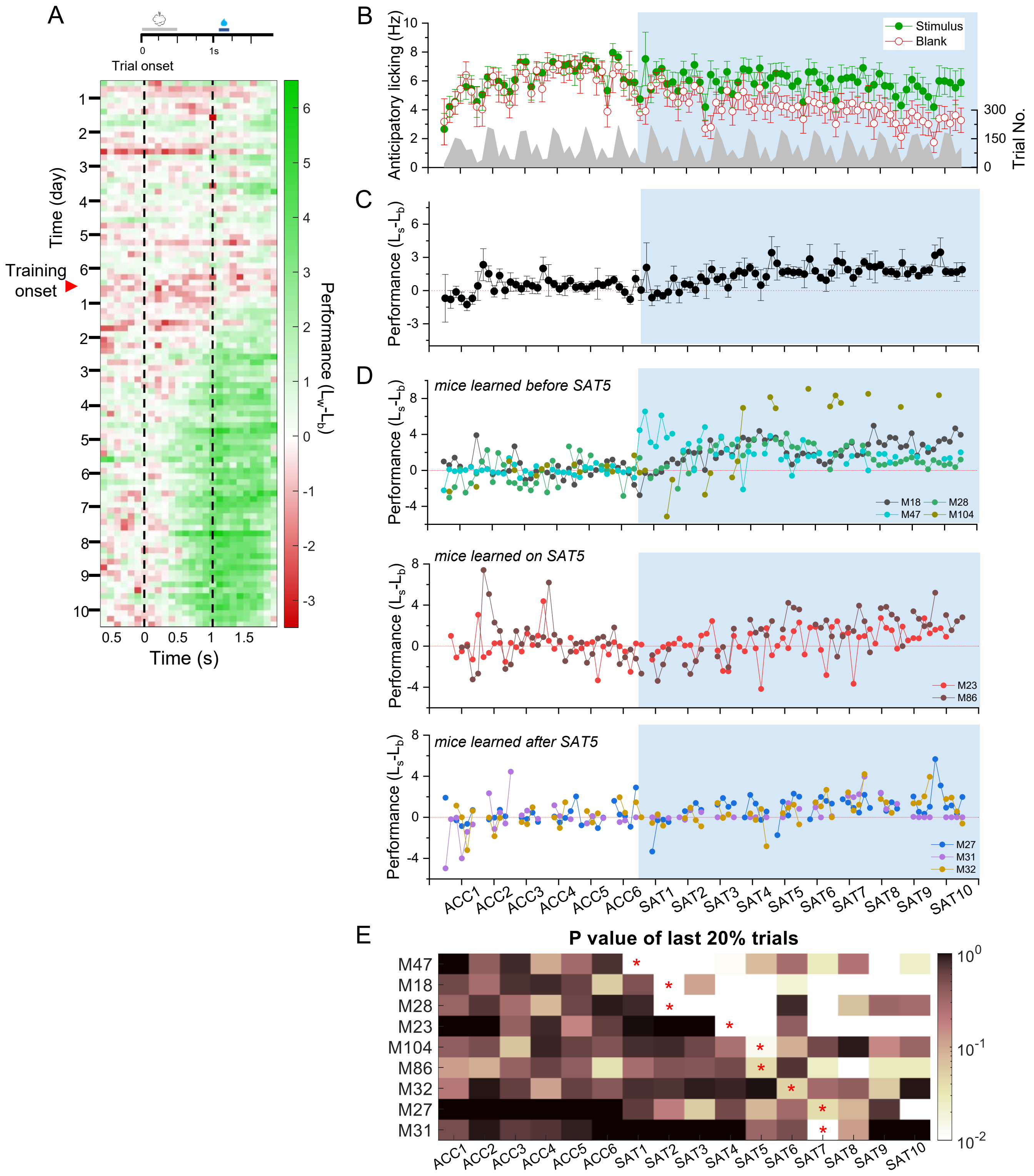

Fig. S1: Anticipatory licking reflects learning during sensory association training (SAT). (**A**) Mean performance performance ($Licking_{stimulus}$-$Licking_{blank}$) averaged across 9 animals. (**B**) Mean anticipatory licking frequency averaged across 9 mice on stimulus (green) and blank (red) trials. (**C**) Mean performance averaged across 9 mice. (**D**) Top: Performance of individual mice learned before SAT5. Middle: same as top, but for mice learned on SAT5. Bottom: Same as top, but for mice learned after SAT5. (**E**) P values of anticipatory licking frequency of the last 20% of stimulus and blank trials for a given training day, for each animal. Asterisks indicate the "learning" day (see methods: criteria for learning).

Fig. S2. Post-hoc imaging location for SST-Cre x Ai148 mice after SAT or PSE

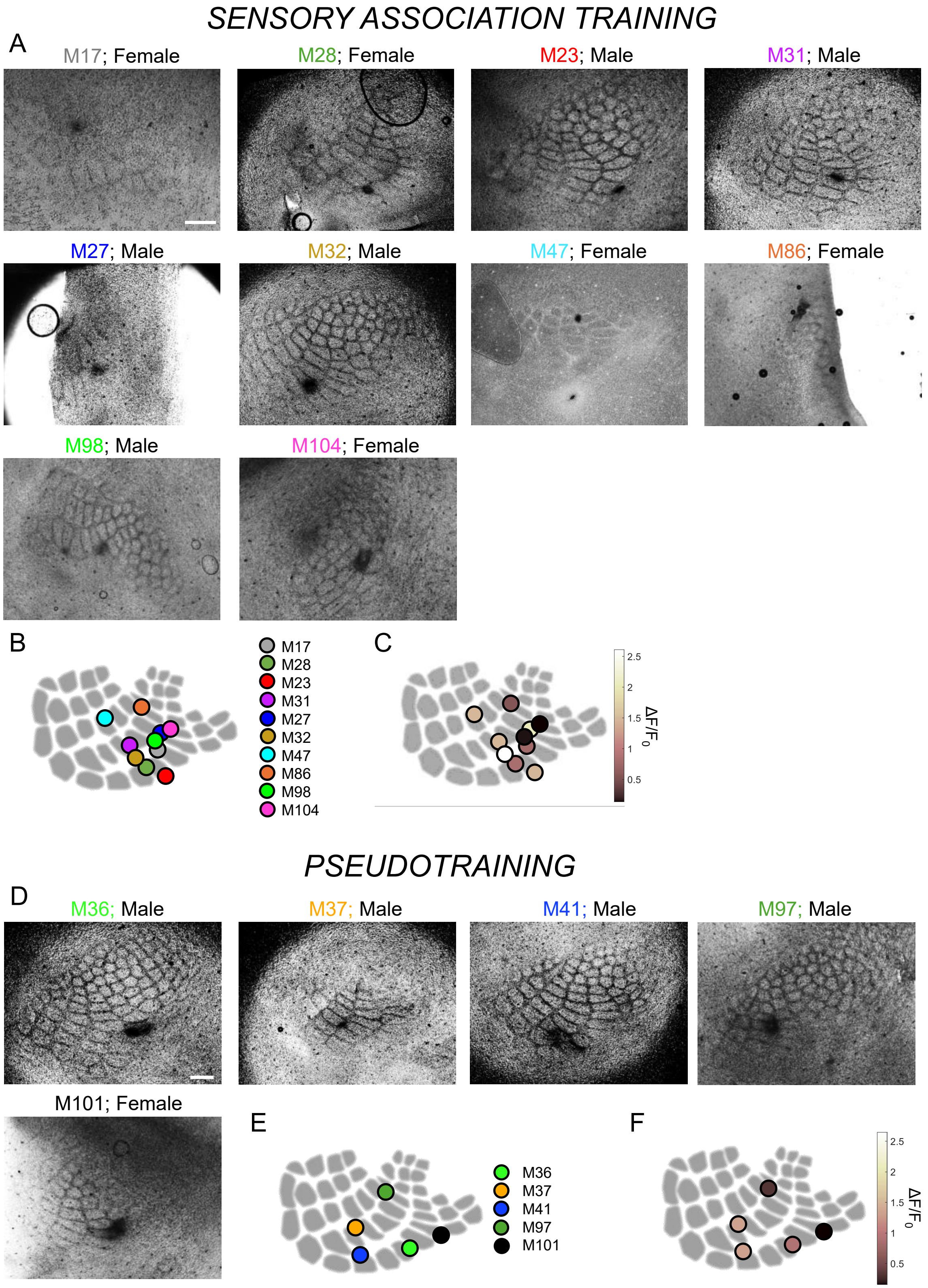

Fig. S2: Post-hoc imaging location for SST-Cre x Ai148 mice after SAT or PSE (**A**) Post-hoc labeling of imaging site with a glass pipette filled with methyl blue dye. Scale=500 µm. (**B**) Schematic diagram of labeled imaging sites of all mice. Each dot represents a mouse. (**C**) Schematic diagram of labeled imaging sites of all mice color coded based on the mean peak response on ACC4-6. (**D-F**) Same is in (A-C) but for pseudotrained animals.

Fig. S3. Stimulus-reward decoupling during pseudotraining (PSE) does not change anticipatory licking

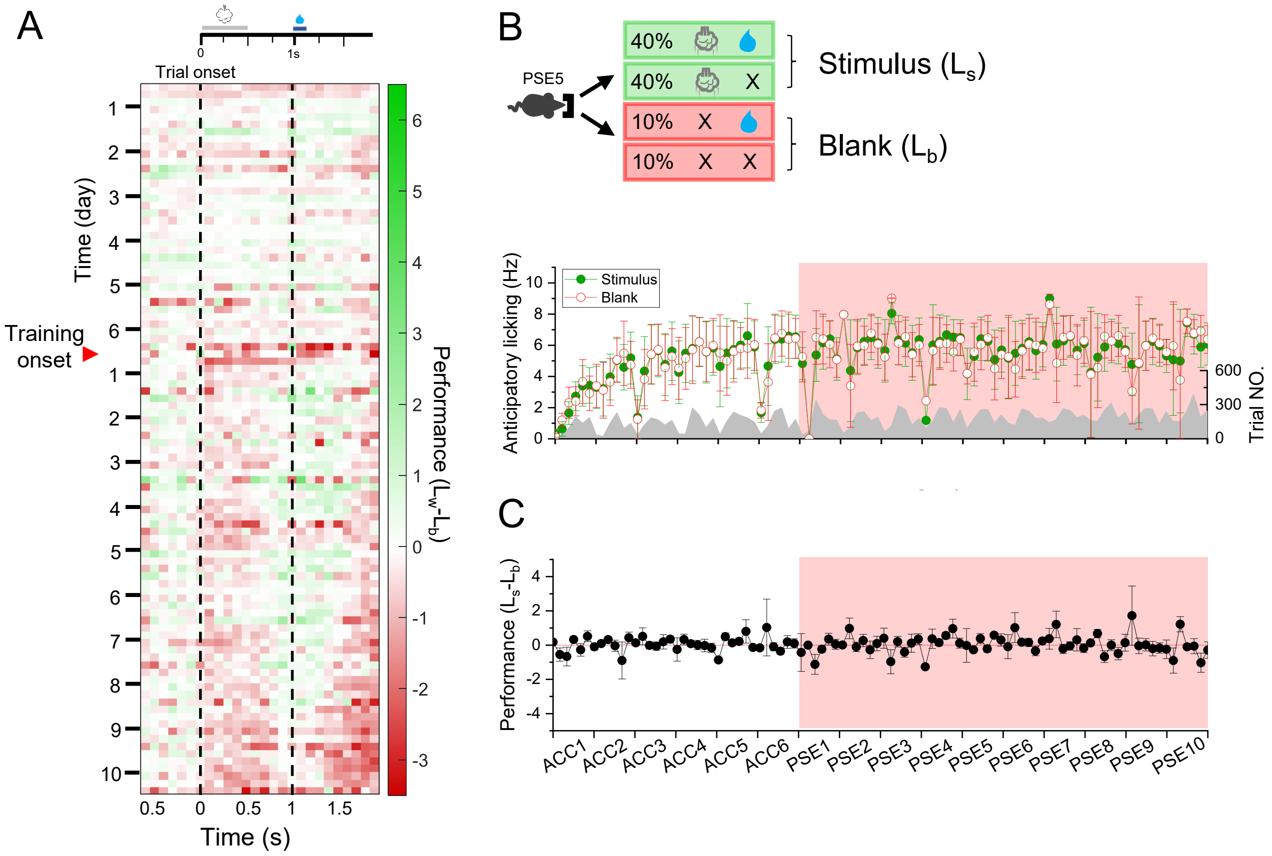

Fig. S3. Stimulus-reward decoupling during pseudotraining (PSE) does not change anticipatory licking. (**A**) Mean performance performance ($Licking_{stimulus}$-$Licking_{blank}$) averaged across 10 animals. (**B**) Top: pseudotraining structure. Bottom: Mean anticipatory licking frequency averaged across 10 mice on stimulus (green) and blank (red) trials. (**C**) Mean performance averaged across 9 mice.

Fig. S4. SST response plasticity outside of S1 barrel cortex

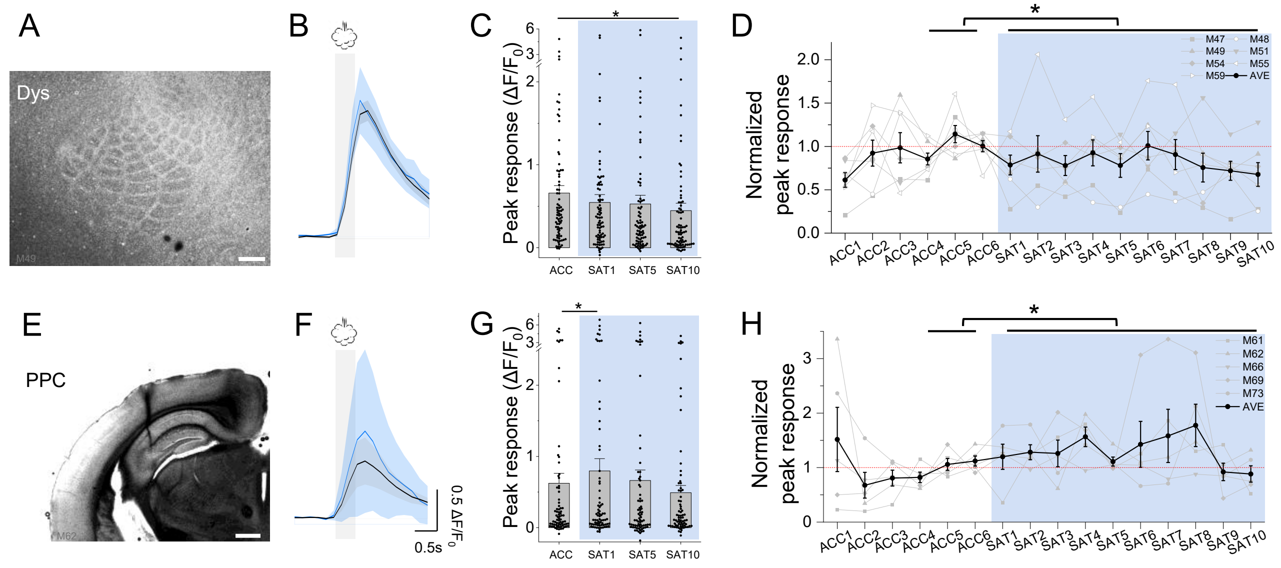

Fig. S4. SST response plasticity outside of S1 barrel cortex. (**A**) Example imaging location in dysgranular zone. Scale bar = 500 μm. (**B**) Averaged trace of the airpuff-evoked response during acclimation 4-6 (ACC4-6) and SAT5. Trace was averaged across 7 mice. Mean ± SEM of shown in the figure. Grey shade indicate the airpuff period. (**C**) Peak evoked response on ACC4-6, SAT1, SAT5, and SAT10. Paired t-test with Bonferroni correction, comparing ACC4-6 with training days, p=0.17, 0.25, and 0.0076 for SAT1, SAT5, and SAT10, respectively. (**D**) Peak airpuff-evoked response across the acclimation and training period, averaged across mice and normalized to ACC4-6. One-way repeated measures ANOVA, p=0.0076. (**E**) Example imaging location in posterior parietal cortex. Scale bar = 0.2 mm. (**F**) Averaged trace of the airpuff-evoked response during acclimation 4-6 (ACC4-6) and SAT5. Trace was averaged across 5 mice. (**G**) Peak evoked response on ACC4-6, SAT1, SAT5, and SAT10. Paired t-test with Bonferroni correction, comparing ACC4-6 with training days, p=0.013, 0.99, and 0.17 for SAT1, SAT5, and SAT10, respectively. (**H**) Peak airpuff-evoked response across the acclimation and training period, averaged across mice and normalized to ACC4-6. One-way repeated measures ANOVA, p=0.013.

Fig. S5. SST response plasticity in males and female mice

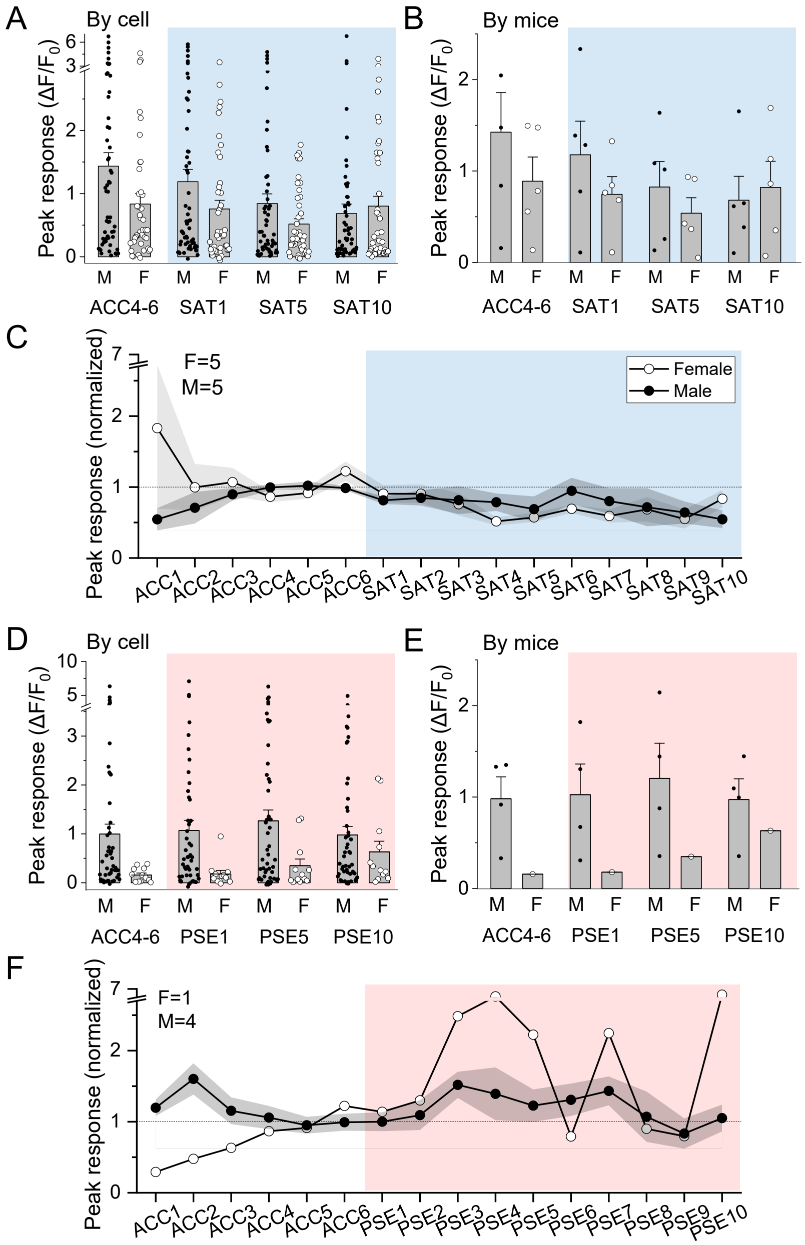

Fig. S5. SST response plasticity in males and female mice. (**A**) Peak evoked response on ACC4-6, SAT1, SAT5, and SAT10 by sex, cell average. M: n=56, F: n=42. (**B**) Peak evoked response on ACC4-6, SAT1, SAT5, and SAT10 by sex, animal average. M: N=5, F: N=5. (**C**) Peak airpuff-evoked response across the acclimation and training period, averaged across mice, grouped by sex, and normalized to ACC4-6. Mean ± SEM of shown in the figure. (**D**) Peak evoked response on ACC4-6, PSE1, PSE5, and PSE10 by sex, cell average. M: n=50, F: n=12. (**E**) Peak evoked response on ACC4-6, , PSE1, PSE5, and PSE10 by sex, animal average. M: N=4, F: N=1. (**F**) Peak airpuff-evoked response across the acclimation and pseudotraining period, averaged across mice, grouped by sex, and normalized to ACC4-6. Mean ± SEM of shown in the figure.

Fig. S6. Tracking response plasticity in individual SST neurons during SAT

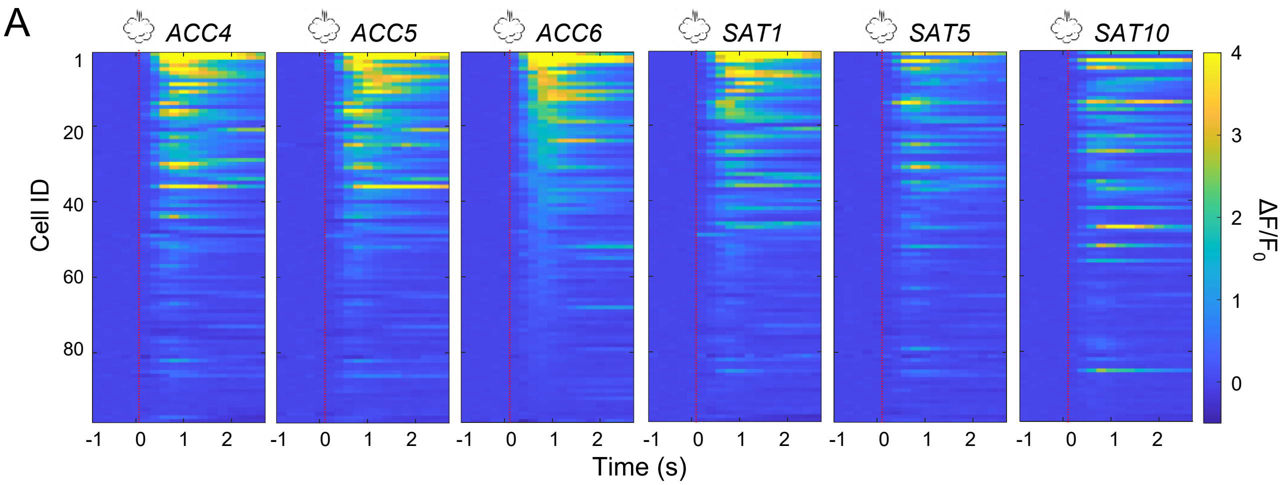

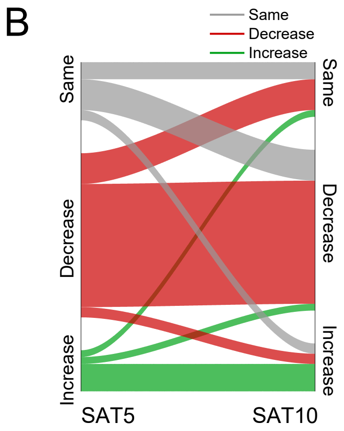

Fig. S6. Tracking response plasticity in individual SST neurons during SAT. (**A**) Evoked response traces of all cells rank ordered by peak response from ACC6, where cell identity is maintained along the y-axis across days. Red dotted lines indicate airpuff onset. (**B**) Sankey plot showing changes in neural activity across sessions on SAT5 (left) to SAT10 (right). Categories are Same (gray; SAT5 n=17 and SAT10 n=16), Decrease (red; SAT5 n=48 and SAT10 n=47), and Increase (green; SAT5 n=12 and SAT10 n=14). Line thickness indicates the proportion of neurons transitioning between states on these two imaging days.

Fig. S7: Intrinsic excitability of L2/3 SST neurons is not altered by SAT

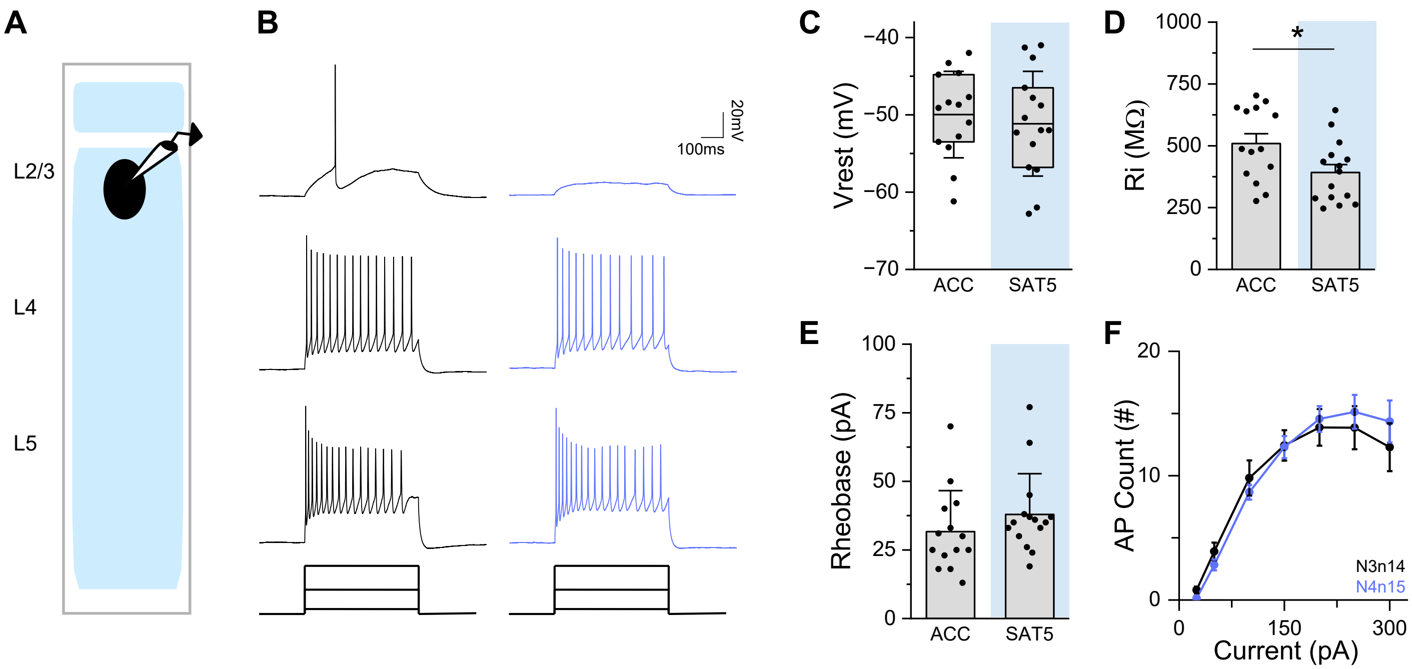

Fig S7 Intrinsic excitability of L2/3 SST neurons during SAT. (**A**) Schematic of the experiment setup. ChR2-expressing SST neurons were targeted. (**B**) Representative firing response following 500 ms 25 pA, 150 pA, and 300 pA current injection recorded from L2/3 low-threshold spiking SST neurons after 6 days of acclimation (ACC6: black) and 5 days of SAT (SAT5: blue). (**C**) Resting membrane potential (Vrest) comparison. ACC6 (-50.0 ± 5.6 mV; N = 3 mice, n = 14 cells) vs. SAT5 (-51.1 ± 6.8 mV; N = 4 mice, n = 15 cells). Box is 25th and 75th quartile, whiskers are SD, and midline is mean. Mann-Whitney U test (U = 115, n = 14 and 15, two-tailed). (**D**) Input resistance (Rin) comparison (mean + SEM). ACC6 (509.1 ± 39.8 MΩ; N = 3 mice, n = 14 cells) vs. SAT5 (391.8 ± 32.0 MΩ; N = 4 mice, n = 15 cells), p=0.03. Mann-Whitney U test (U = 155, n = 14 and 15, two-tailed). (**E**) Rheobase comparison (mean + SD). ACC6 (31.6 ± 15.0 pA, N = 3 mice, n = 14 cells) vs. SAT5 (37.9 ± 14.8 pA, N = 4 mice, n = 15 cells). Mann-Whitney U test (U = 70.5, n = 15 and 14, two-tailed). (**F**) F-I curve of L2/3 SST neurons in ACC6 (black) and SAT5 (blue) animals. Mean ± SEM. Two-way repeated measures ANOVA $F_{(1,27)} = 0.01$, p = 0.91.

Fig. S8. Analysis pipeline for PSD95-FingR puncta reconstruction and dendritic localization

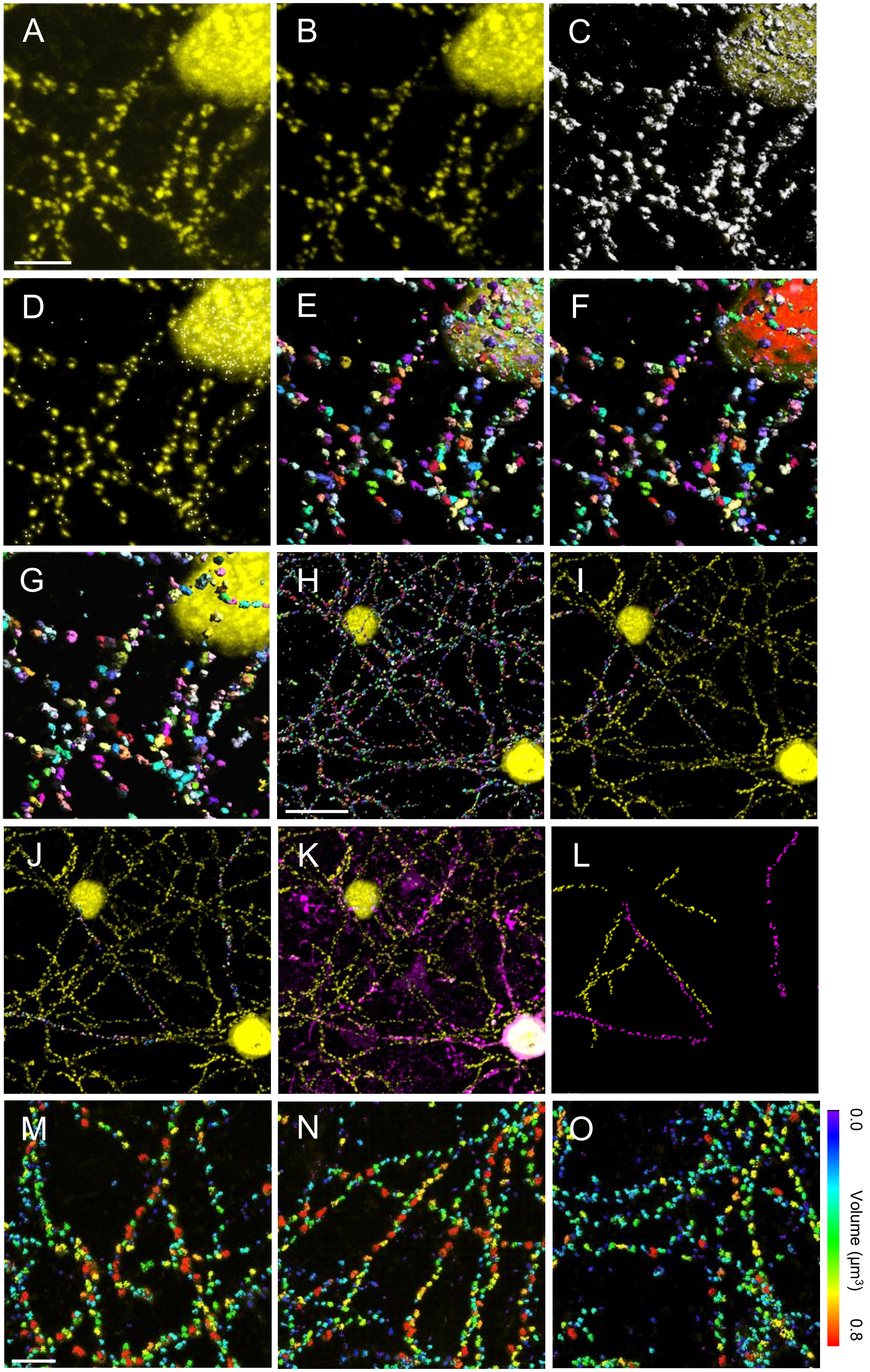

Fig. S8. Analysis pipeline for PSD95-FingR puncta reconstruction and dendritic localization. (**A**) Region of interest (ROI) containing raw PSD95.FingR signal in confocal image stack (63x). Scale=5μm (**B**) Background subtraction prior surface reconstructions. (**C**) Semi-automated thresholding of 3D surface masks (gray) to optimize signal coverage while minimizing noise contained in background voxels. (**D**) A quality filter designating seed points (punctate signal) for splitting fused 3D surface masks. (**E**) 3D reconstructed fluorescence signal (multi-colored) after filtering structures to be ≥ 3 voxels to visualize all PSD95.FingR surface objects. (**F**) 3D surface mask of somatic PSD95.FingR signal. (**G**) 3D surface masks of PSD95.FingR signal within 0.15 um from reconstructed soma were removed. Remaining punctate PSD95.FingR signal (multi-colored) were included for whole field puncta analysis. (**H**) Zoomed out ROI from the same confocal image stack containing punctate PSD95.FingR signal for single cell puncta localization and subsequent comparisons between SST subtypes. Scale=15μm (**I**) Manually selected 3D surfaces (multi-colored) belonging to traceable dendrites emanating from soma in the top left corner. (**J**) Same as (**I**) but for soma in the bottom right corner. (**K**) Calb2-IR (purple) revealed for classifying puncta belonging to SST-Calb2 or SST-O. (**L**) Designated puncta belonging to SST-O (yellow) and SST-Calb2 (purple) neurons. (**M-O**) A zoomed 63x image stack visualizing puncta volume in ACC, SAT1, and SAT5 respectively. Smaller puncta are represented with cool colors while larger puncta are represented with warm colors. Scale bar=10μm. Volume scale 0-0.8 um^3

Fig. S9. Field-of-view analysis of PSD95 puncta indicates layer-specific regulation of excitatory synapses onto SST neurons

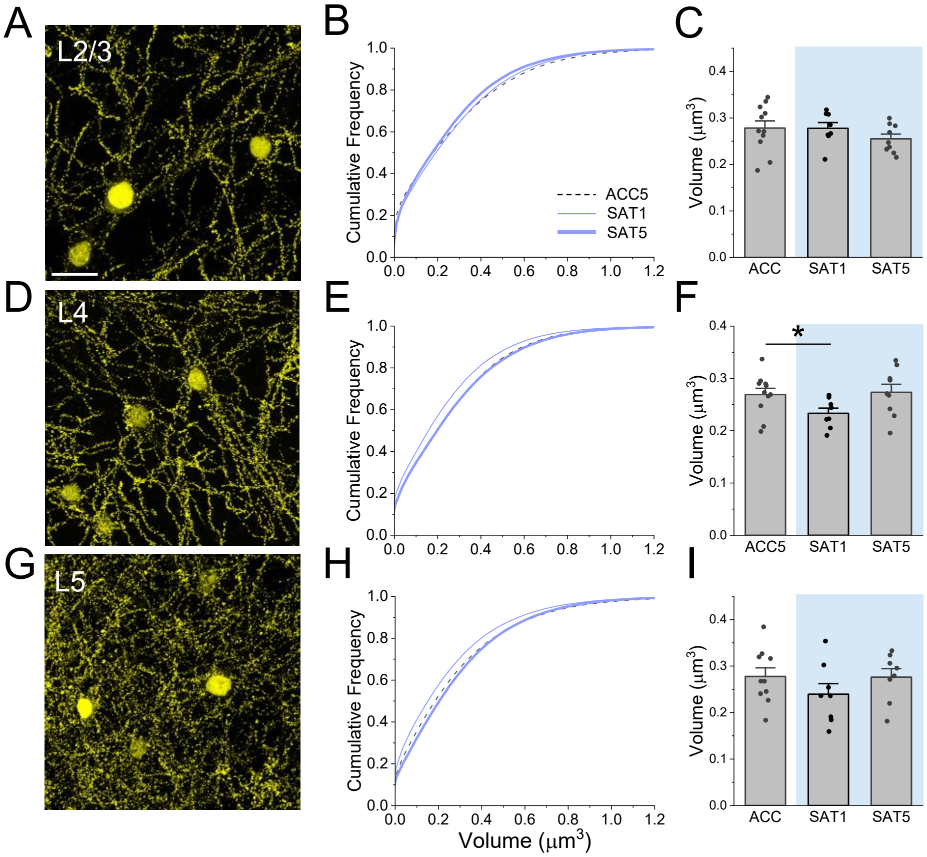

Fig. S9. Field-of-view analysis of PSD95 puncta indicates layer-specific regulation of excitatory synapses onto SST neurons. (**A**) Representative field of view from PSD95.FingR labeled SST neurons in L2/3 of primary somatosensory cortex. Scale=20µm. (**B**) Cumulative distribution of reconstructed PSD95.FingR surface volume from whole field analysis in acclimated (black dashed line), one day (thin blue line), and five day trained (thick blue line) SST-Cre mice. (**C**) Mean puncta volume in individual animals across training conditions. (ACC N=11 mice, 60,500 puncta; SAT1 N=8, 44,000 puncta; SAT5 N=9, 49,500) puncta (**D-F**) Same as A-C but for L4 PSD95.FingR labeled SST neurons. (ACC N=11 mice, 79,420 puncta; SAT1 N=8, 57,760 puncta; SAT5 N=9, 64,980 puncta; ACC vs SAT1 p=.04, unpaired t-test) (**G-I**) Same as A-C but for L5 PSD95.FingR labeled SST neurons. (ACC N=11 mice, 98,900 puncta; SAT1 N=8, 79,120 puncta; SAT5 N=9, 89,010 puncta).

Fig. S10. SAT does not differentially suppress SST-Calb2 neurons in V1.

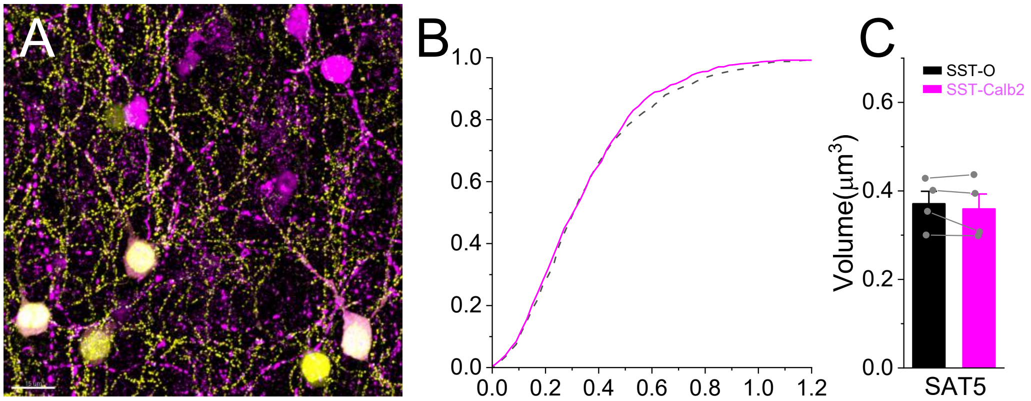

Fig. S10. SAT does not differentially suppress SST-Calb2 neurons in V1. (**A**) 63x confocal image stack containing PSD95.FingR labeled SST neurons merged with calretinin-IR from L2/3 of V1. Scale = 20μm (**B**) Cumulative distribution of PSD95 puncta volume in SST-O and SST-Calb2 neurons (SST-O N = 4mice, 800 puncta; SST-Calb2 N = 4 mice, 800 puncta; Ks test: p=0.34). (**C**) Within animal comparison between L2/3 V1 SST-O neurons (black bars) and SST-Calb2 (pink bars) during acclimation or 5 days of SAT.

Fig. S11. Neither pseudotraining nor sensory enrichment induce subtype-specific changes in L2/3 SST neurons.

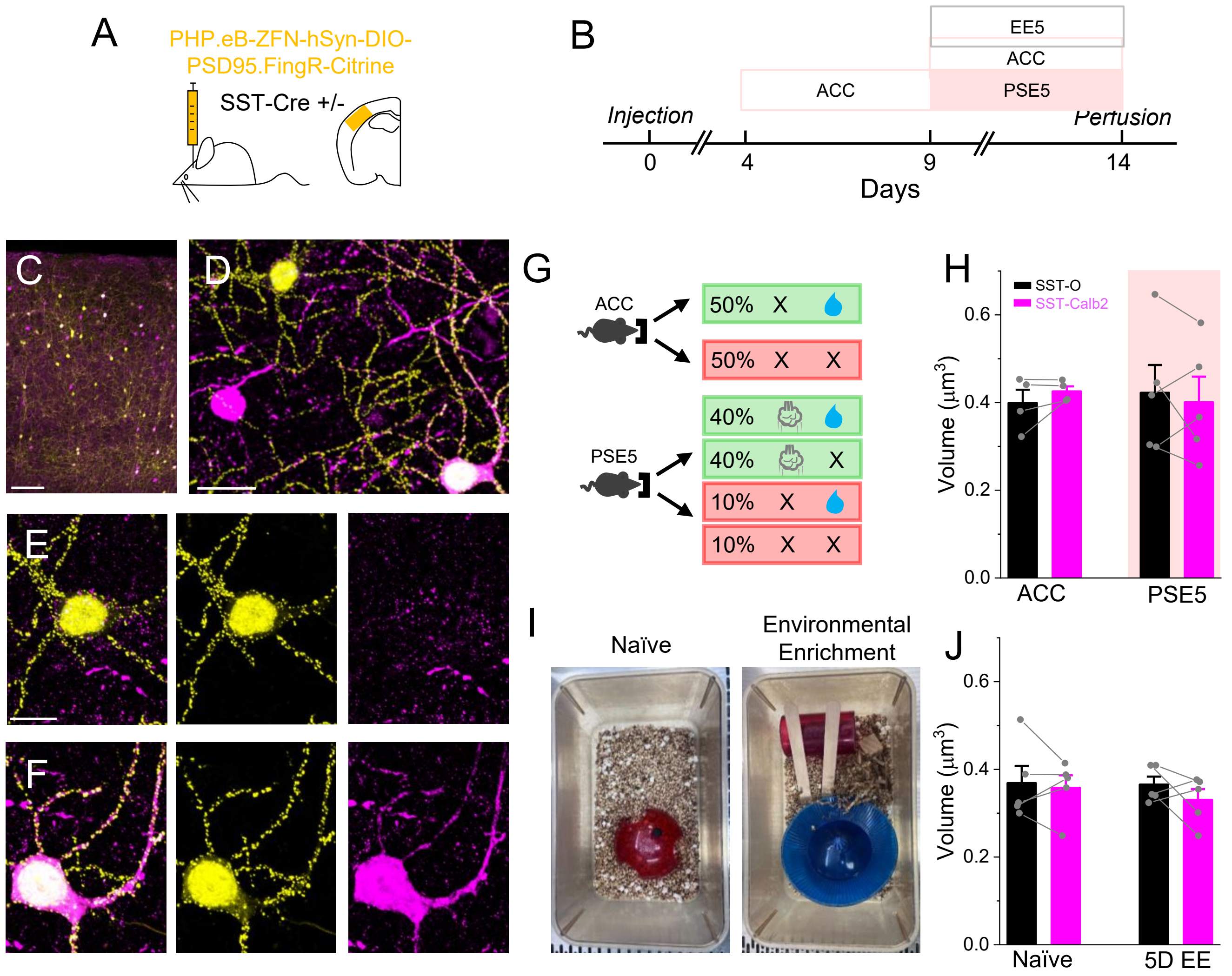

Fig. S11. Neither pseudotraining nor sensory enrichment induce subtype-specific changes in L2/3 SST neurons. (**A**) Schematic outlining viral injection strategy. (**B**) Schematic outlining training timeline. (**C**) 10x image of PSD95.FingR labeled SST neurons merged with calretinin-IR. Scale = 100μm (**D**) 63x confocal image stack containing PSD95.FingR labeled SST neurons merged with calretinin-IR. Scale = 20μm (**E**) Zoomed image of SST-O neuron. PSD95.FingR-Citrine (left), calretinin-IR (middle), overlay, (right). Scale = 5μm (**F**) Same as (E) but for an SST-Calb2 neuron. (**G**) Schematic outlining stimulus and reward presentation probabilities after trial initiations in pseudo-training. (**H**) Within animal comparison between SST-O neurons (black bars) and SST-Calb2 (pink bars) during acclimation or 5 days of pseudo-training (PSE5). (ACC N = 4mice, 800 puncta; PSE N = 5 mice, 1000 puncta). (**I**) Image showing naïve (left) and environmentally enriched cages (right). (**J**) Same as (H) but for naïve animals and animals experiencing five days of environmental enrichment (EE5). (Naive N = 5mice, 1000 puncta; EE5 N = 5 mice, 1000 puncta)

I

Fig. S12. Chemogenetic suppression of SST neuron activity reduces PSD95 puncta size on both SST-O and SST-Calb2 neurons

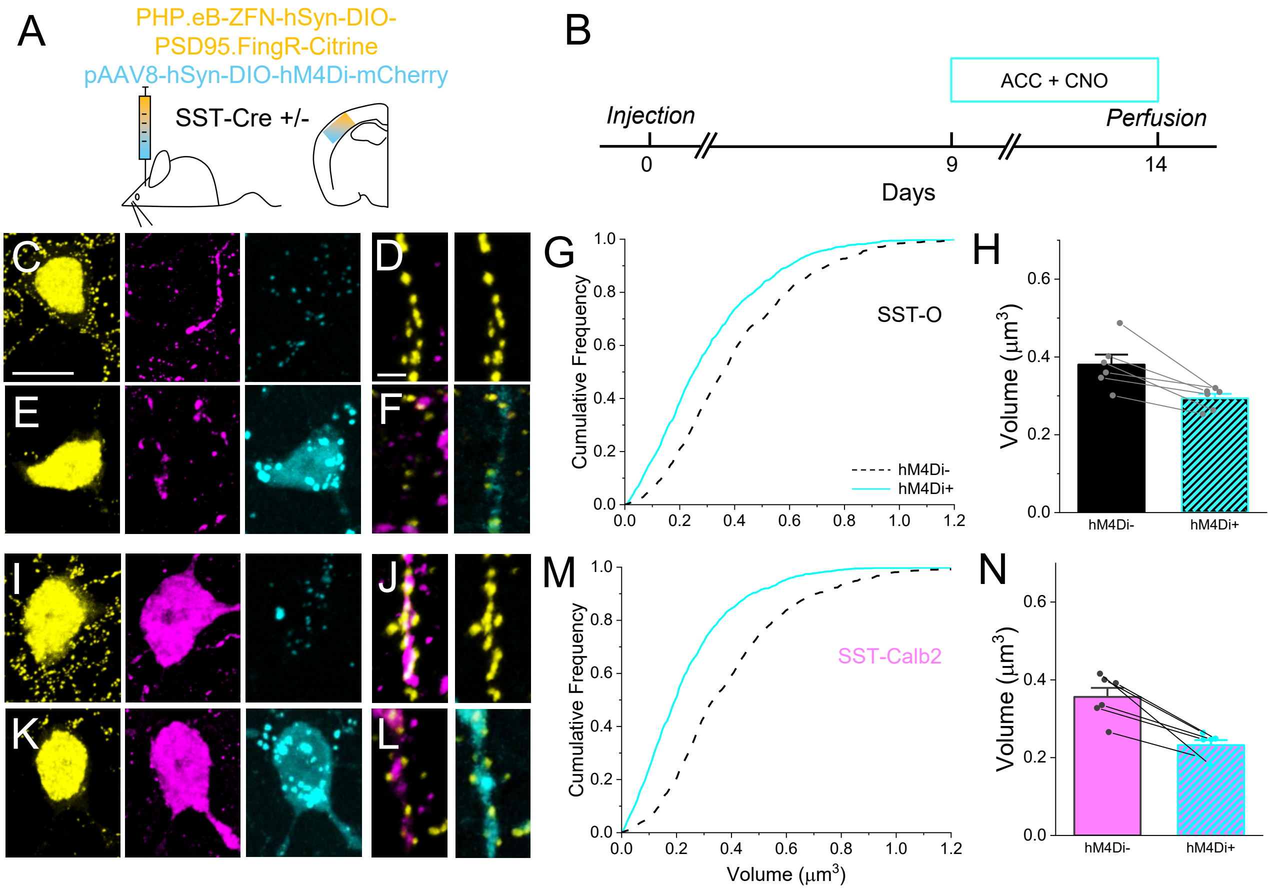

Fig. S12. Chemogenetic suppression of SST neuron activity reduces PSD95 puncta size on both SST-O and SST-Calb2 neurons. (**A**) Schematic of Viral injection. (**B**) Schematic of experimental timeline. (**C**) 63x somatic ROI of volumetric confocal image stack containing from left to right, PSD95.FingR-Citrine (yellow), calretinin-IR (purple), and hM4Di-mCherry (pseudo-colored cyan) fluorescence channels. (**D**) Dendrite example from an SST-O neuron (left) overlay between PSD95.FingR (yellow) and calretinin-IR (purple). (right) overlay between PSD95.FingR and hM4Di-mCherry (cyan). (**E, F**) same as in (C, D) but for an SST-O neuron transduced with hM4Di-mCherry. (**G**) Cumulative distribution of PSD95 puncta volume in SST-O neurons with and without hM4Di expression (hM4Di- N = 6mice, 1200 puncta; hM4Di+ N = 6 mice, 1200 puncta). (**H**) Within animal comparison of average PSD95 puncta size in SST-O cells with and without hM4Di expression. (**I, J**) same as in (C, D) but an SST-Calb2 neuron not transduced with hM4Di-mCherry. (**K, L**) same as in (C, D) but for an SST-Calb2 neuron transduced with hM4Di-mCherry. (**M, N**) Same as in (G,H) but for SST-Calb2 neurons (hM4Di- N = 6mice, 1200 puncta; hM4Di+ N = 6 mice, 1200 puncta).

Fig. S13. Identification of SST-Calb2 neurons and training-dependent responses

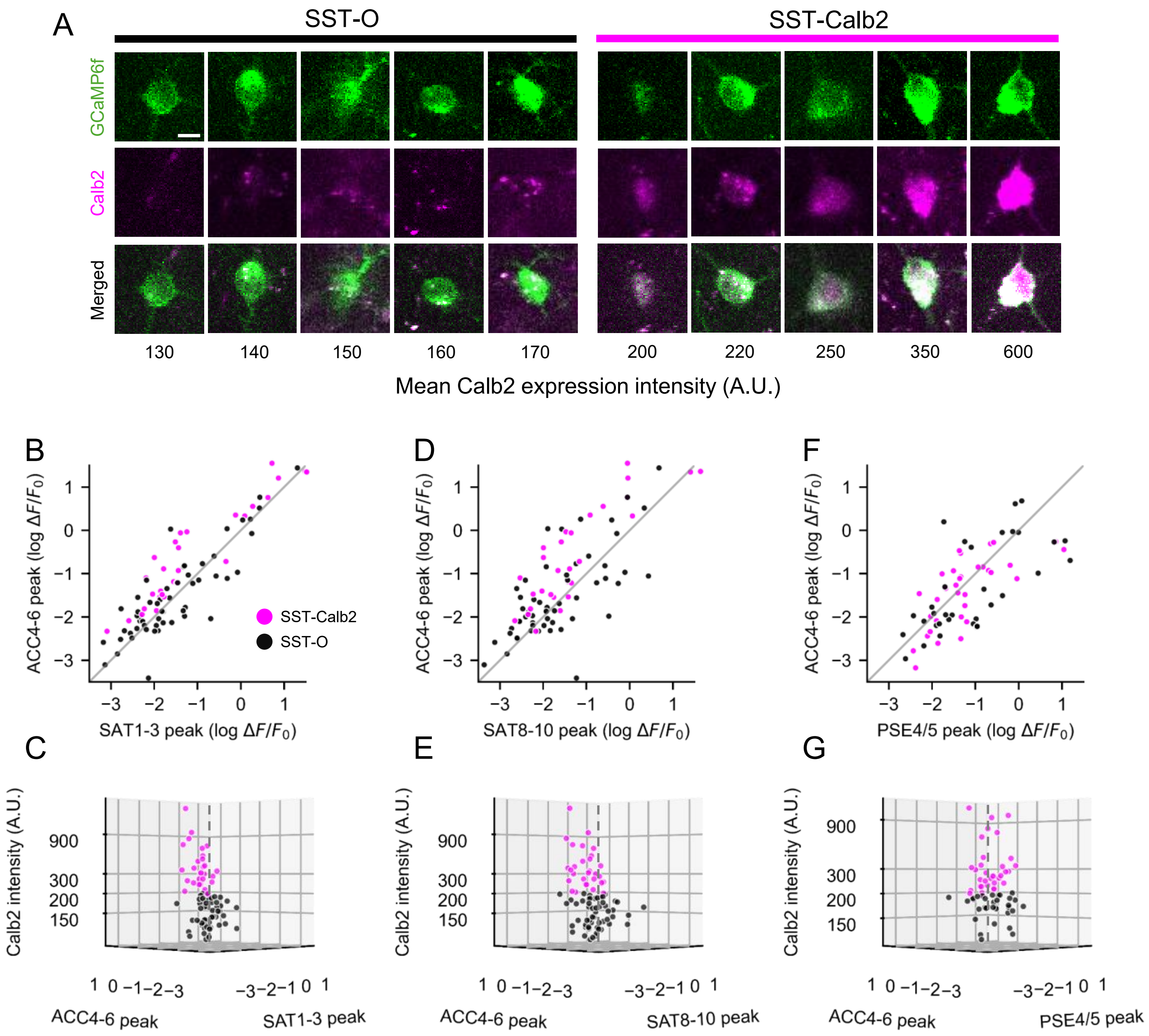

Fig. S13. Identification of SST-Calb2 neurons and training-dependent responses. (A) Example neurons showing GCaMP6f expression (green) and Calb2 expression (magenta), with merged images (bottom) displaying both markers. The neurons are arranged from left to right in increasing order of Calb2 expression. Scale bar=10 μm. (B) Scatterplot of individual cell responses to compare peak stimulus-evoked amplitude between the pretraining (ACC) and early SAT period. Solid black line indicates unity. Note that the SST-Calb2 cells show a leftward shift, indicating decreased responses. n=29 SST-Calb2, 55 SST-O neurons. (C) Scatterplot to show Calb2 fluorescence intensity related to the decrease in activity during the early SAT period, where a fluorescence threshold of 200 A.U. separates the decreasing from stable responsive cells. (D) As in (B-C) but for the late SAT period. (F-G) as in (B-C) but for SST-Calb2 neurons from pseudotrained animals. n=33 SST-Calb2, 33 SST-O.

Fig. S14. Comparison of SST-Calb2 neurons identified by genetic versus immunolabeling in SST-Flp x Calb2-Cre transgenic mice

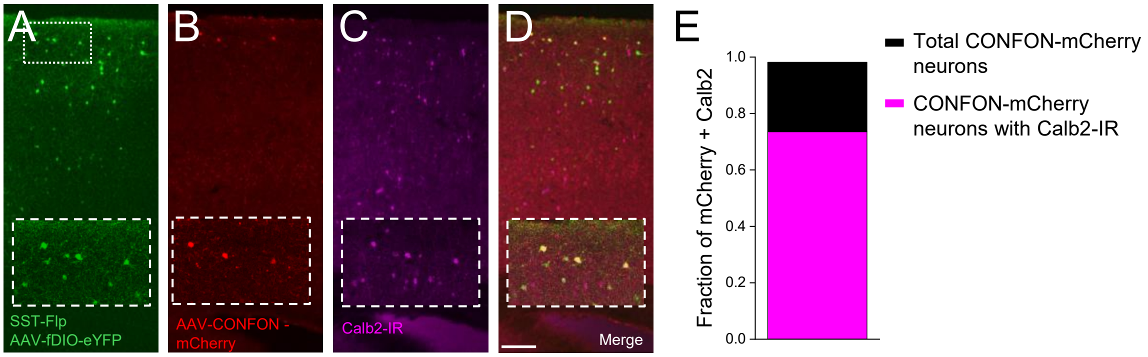

Fig. S14. Comparison of SST-Calb2 neurons identified by genetic versus immunolabeling in SST-Flp x Calb2-Cre transgenic mice. (A) SST-Flp neurons, labeled using a Flp-dependent YFP reporter virus. **(B)** The same tissue, but where SST-Flp, Calb2-Cre neurons are labeled using a Cre-On, Flp-On (CONFON) mCherry reporter virus. **(C)** The same tissue, but using Calb2 immunolabeling. Note that other cortical interneurons (VIP subtype) also express Calb2. **(D)** Merge of **(A-C)** Scale = 50 μm**.** **(E)** Examination of SST-Flp, Calb2-Cre (CONFON-mCherry) positive neurons that are also immunoreactive for Calb2 reveals that 70.7% of CONFON cells also show detectable Calb2 protein (N=3 mice; 68 mCherry-labeled neurons). This indicates that there is a higher threshold for detection of Calb2 protein compared to genetic labeling methods.

Fig. S15. Changes in anticipatory licking in SST-Flp x Calb2-Cre transgenic mice during SAT

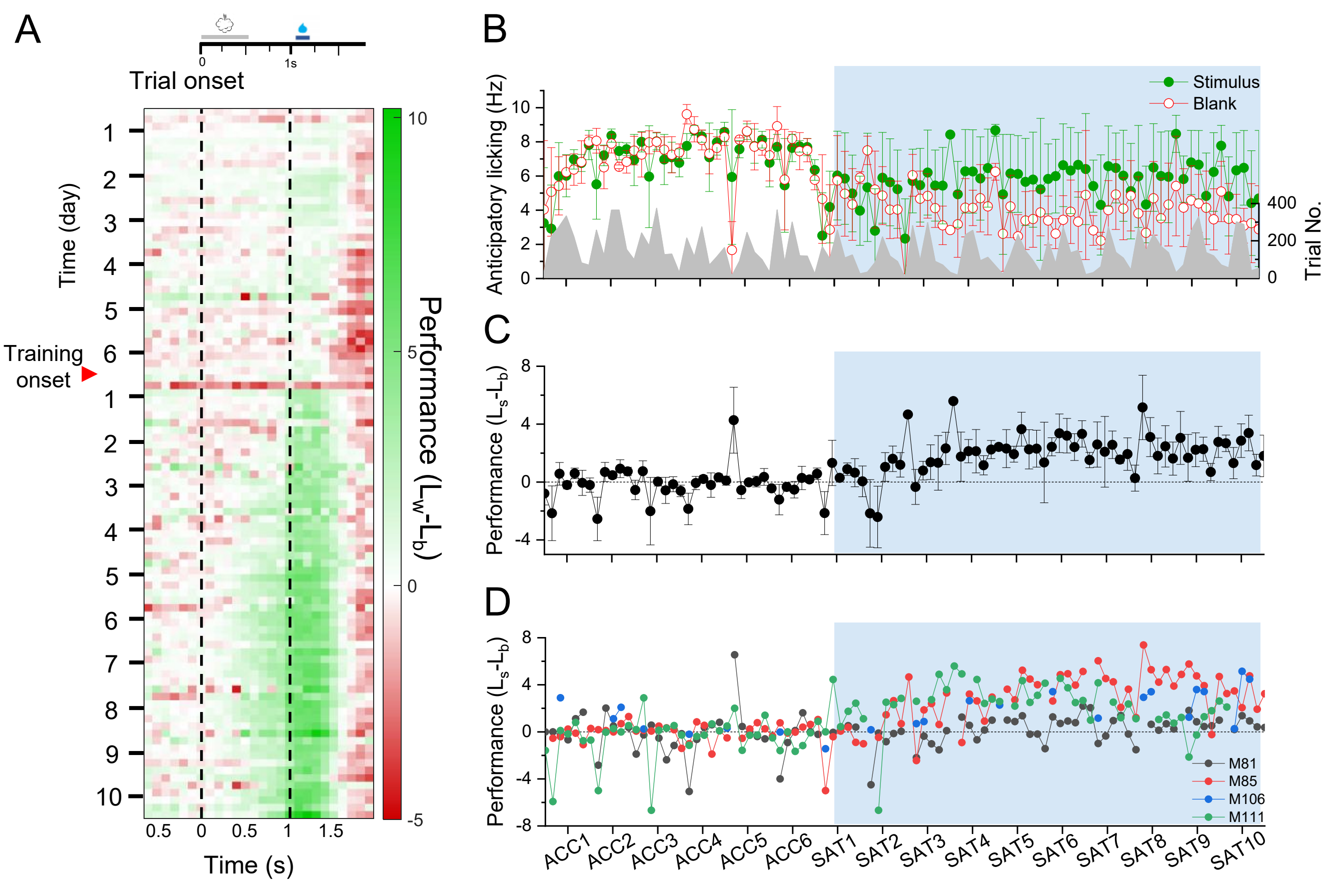

Fig. S15. Changes in anticipatory licking in SST-Flp x Calb2-Cre transgenic mice during SAT. (**A**) Mean performance ($Licking_{stimulus}$-$Licking_{blank}$) averaged across 5 animals. (**B**) Mean anticipatory licking frequency averaged across 4 mice on stimulus (green) and blank (red) trials. (**C**) Mean performance averaged across 4 mice. (**D**) Performance of individual mice.

Fig. S16. Post-hoc imaging location for SST-Flp x Calb2-Cre mice

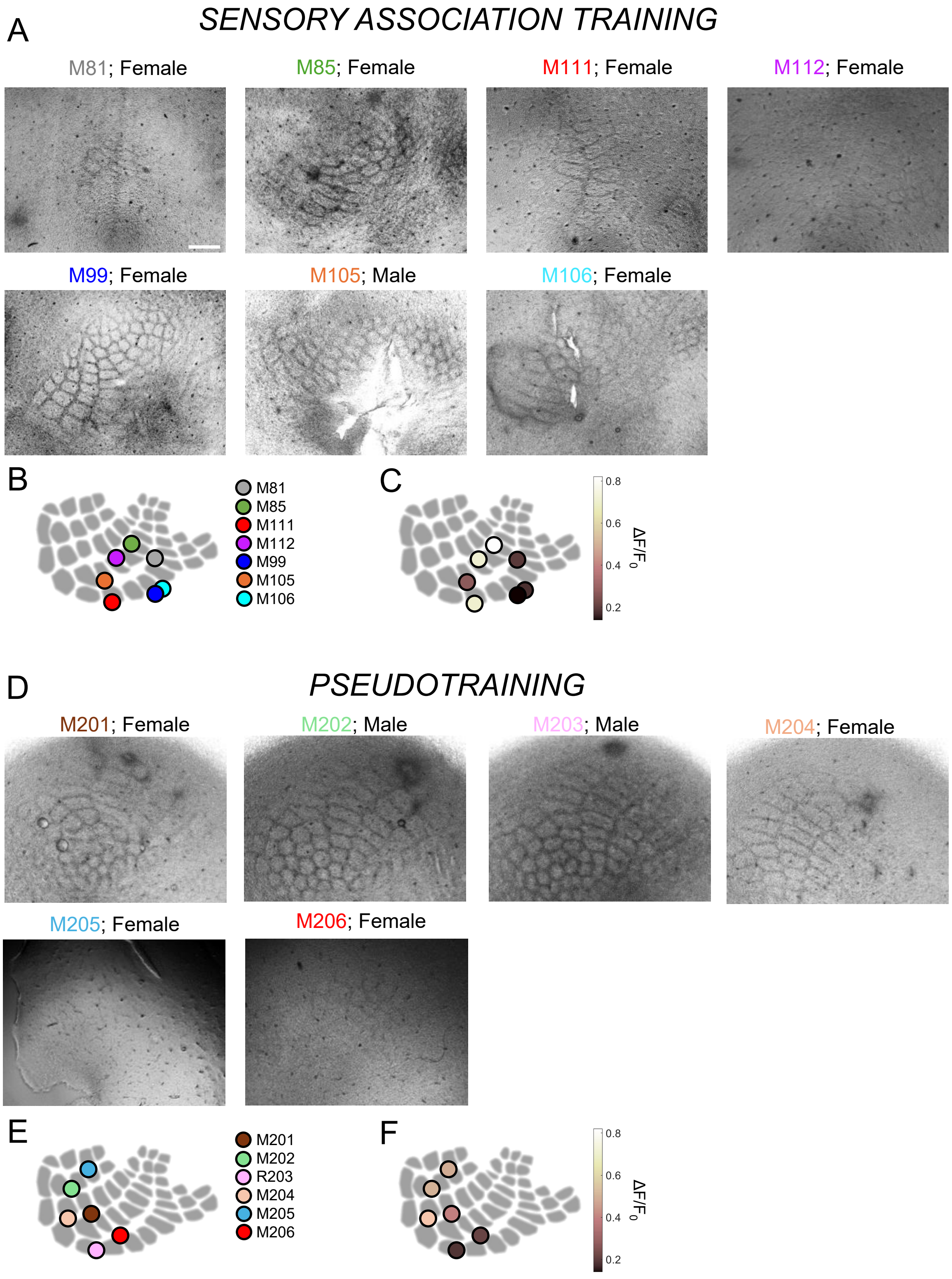

Fig. S16: Post-hoc imaging location for SST-Flp x Calb2-Cre mice after SAT or PSE (**A**) Post-hoc labeling of imaging site for SAT animals using methyl blue dye. Scale=500 µm. (**B**) Schematic diagram of labeled imaging sites of all SAT mice. Each dot represents a mouse. (**C**) Schematic diagram of labeled imaging sites for SAT mice color coded based on the mean peak response on ACC4-6. (**D-F**) Same is in (A-C) but for pseudotrained animals.

Fig S17. SAT specifically reduced evoked response in SST-Calb2 neurons

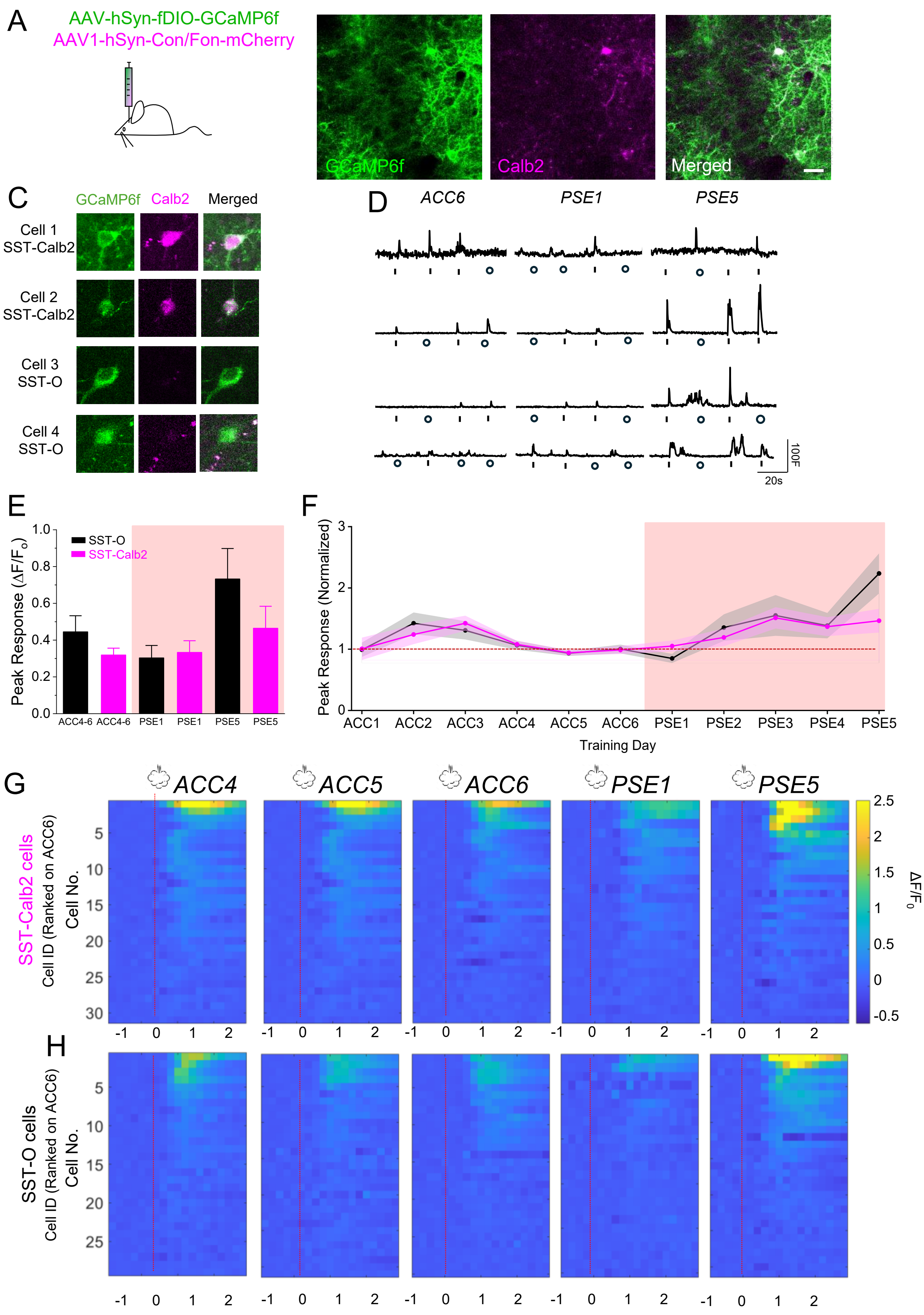

Fig S17. PSE Training does not alter stimulus evoked response A) Schematic demonstrating the stereotaxic delivery of viral constructs into mouse barrel cortex. (**B**) Example FOV expressing GCaMP6f in SST cells and mCherry in Calb2-positive cells. Scale bar = 20 µm. (**C**) Left: example SST-Calb2 and SST-O neurons showing GCaMP6f expression (green) and Calb2 expression (magenta), with merged images (bottom) displaying both markers. (**D**) Example traces of example SST-Calb2 and SST-O cells on ACC6, PSE1, and PSE5 (**E**) Mean peak response across ACC1-6 and PSE1-5. The magenta line represents the average peak response across 33 SST-Calb2 cells while the black line represents the average peak response across 33 SST-O cells collected in 6 mice. One-way repeated measures ANOVA, $p=1.23x10^{-8}$ for SST-Calb2 and $p=4.21x10^{-8}$ for SST-O. (**F**) Mean peak response of SST-Calb2 and SST-O cells on ACC4-6, PSE1, and PSE5. Paired t-test with Bonferroni correction, comparing between SST-Calb2 and SS-O cells within different imaging days, on ACC4-6, PSE1, and PSE5 respectively(p=0.23, p=0.74, p=0.24). (**G**) Response heatmaps of SST-Calb2 rank ordered on ACC6. The red line indicates in the onset of airpuff. (**H**) As in (G), but for SST-O cells.

Fig. S18. Example whole-session Ca++-imaging of L2/3 SST neurons

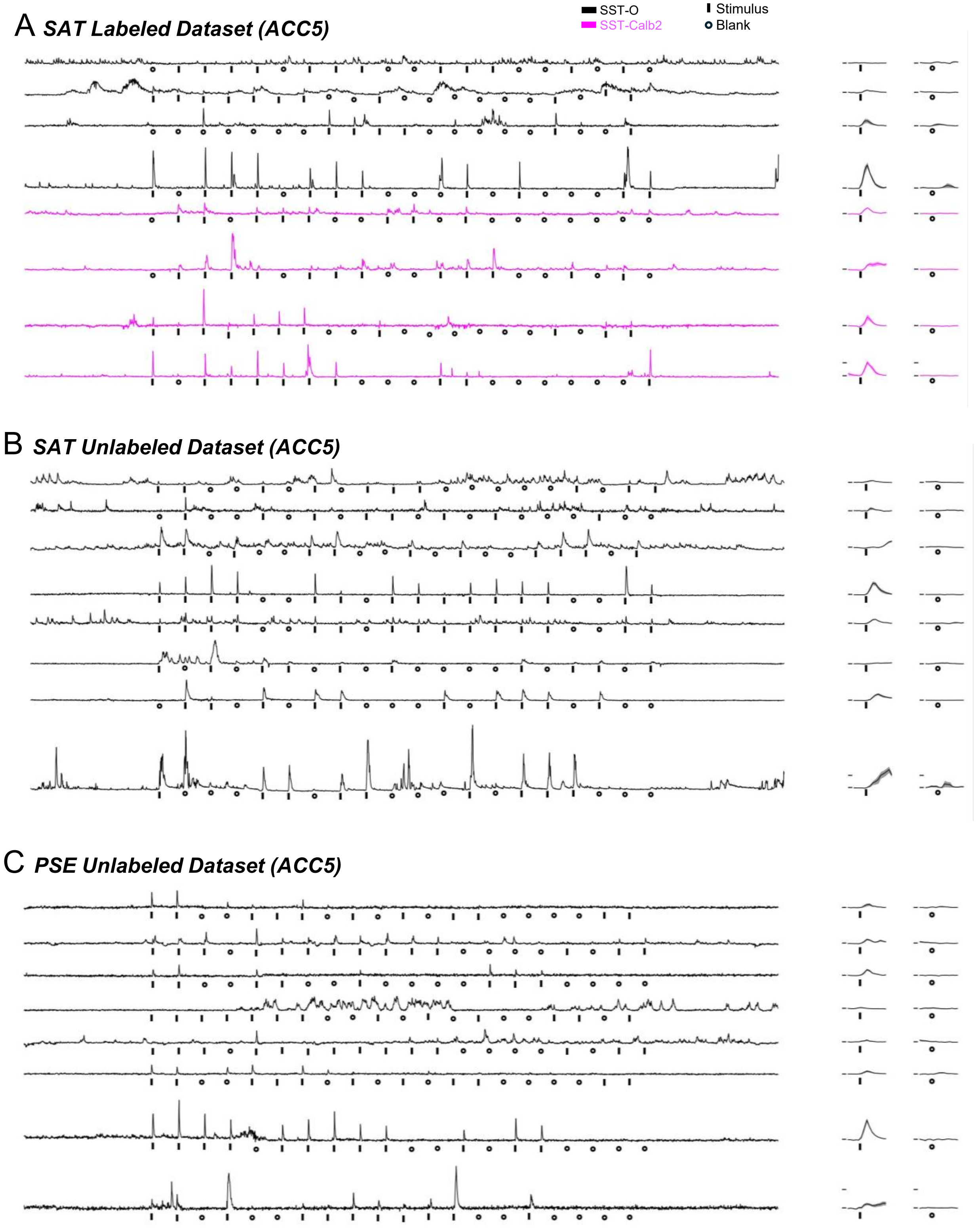

Fig. S18. Example whole-session Ca^{++}-imaging of L2/3 SST neurons. (**A**) Whole-session ACC5 example cells for SST-Flp x Calb2-iCre SAT dataset. (**B**) As in (A), but for SST-Cre x Ai148 SAT dataset. (**C**) As in (A), but for SST-Cre x Ai148 PSE dataset.

Fig. S19. Feature extraction pipeline from Ca++ signals

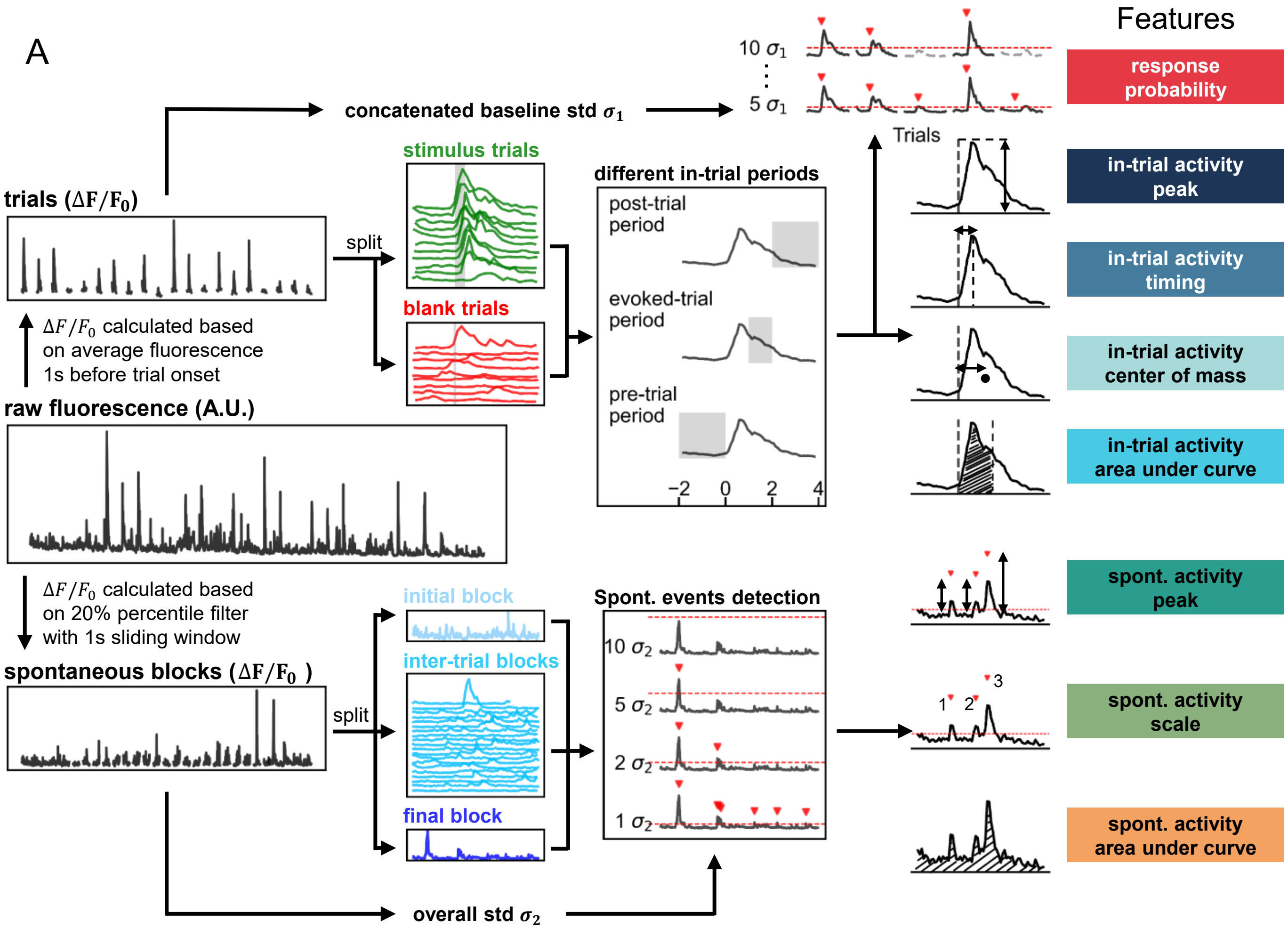

Fig. S19. Feature extraction Pipeline. (**A**) Features for clustering were derived from raw fluorescence and categorized into three main groups: response probability, in-trial measurements, and spontaneous activity measurements. These features were calculated using various parameters, including different timing periods, trial types, spontaneous block types, and thresholding methods. The full feature space encompasses all possible combinations of these parameter setups. Thresholds for response probability features (σ_1) and spontaneous event detection (σ_2) were calculated differently. Specifically, σ_1 (for responsive trial detection) was determined from the standard deviation of concatenated baseline periods (1s before trial onset) from all trials. In contrast, σ_2 (for spontaneous activity detection) was the overall standard deviation of the entire session's fluorescence trace.

Fig. S20. Selection and characteristics of top features for cell clustering

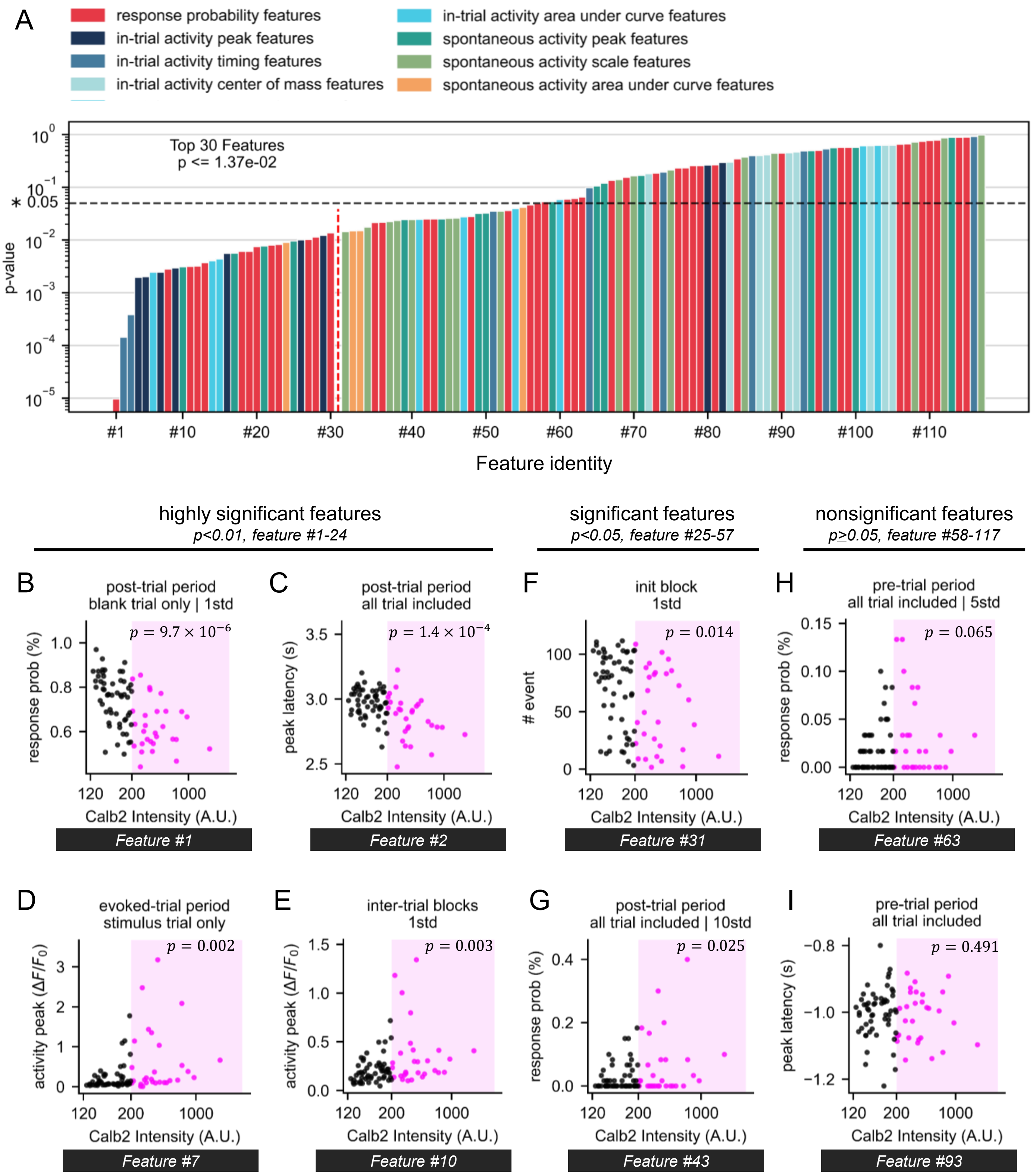

Fig. S20. Selection and characteristics of top features for cell clustering. (**A**) Bar graph ranking features by p-value from unpaired t-test comparing SST-Calb2 and SST-O cell feature averages. The top 30 features (highlighted) were selected for clustering, with representative clusters chosen based on Silhouette score and consistency with transcriptomic hypotheses. (**B-I**) Scatter plots of randomly selected example features compared with Calb2 intensity. The number in the black box indicates the feature's rank from (A).

Fig. S21. Justification for number of clusters using heuristic evaluation

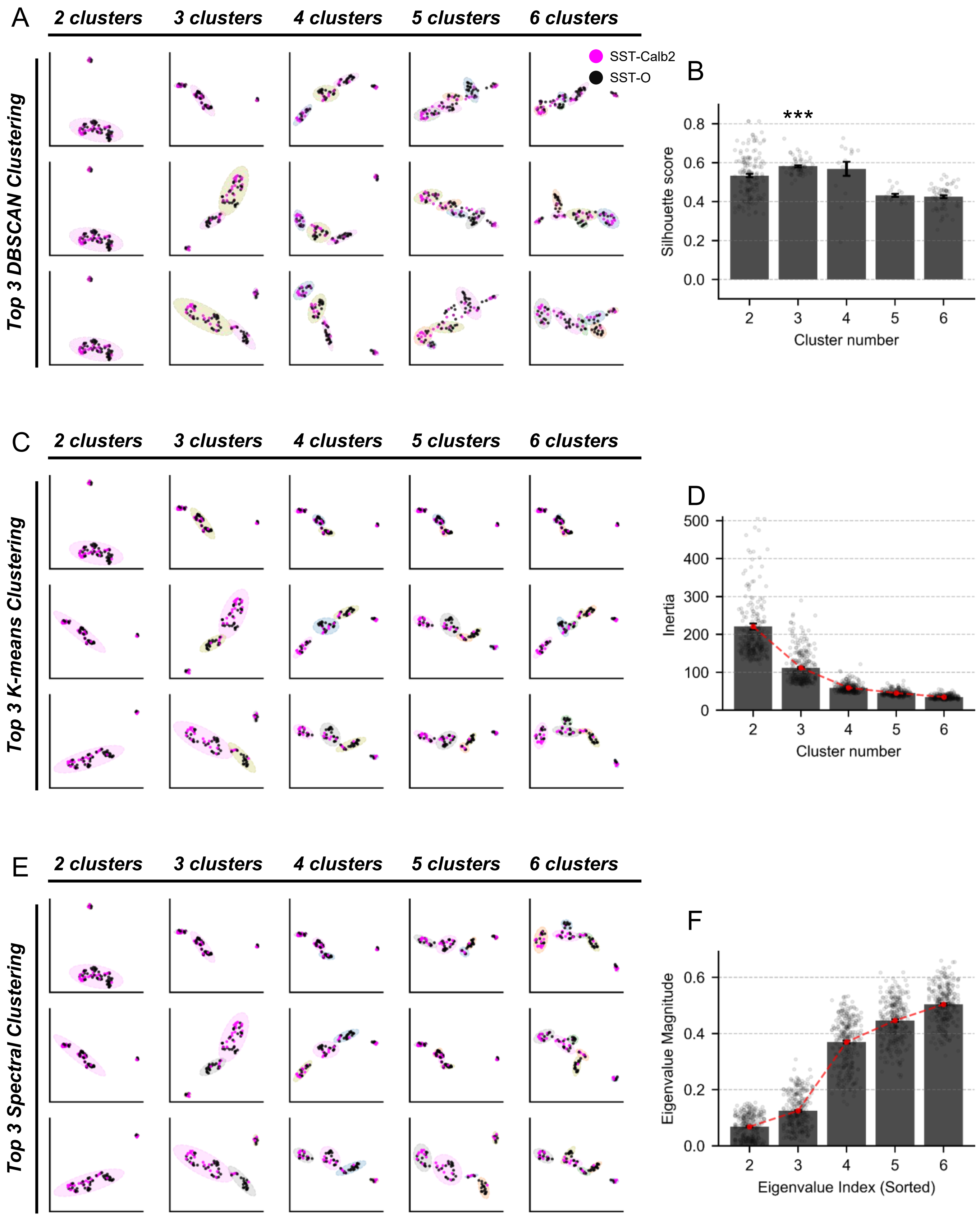

Fig. S21. Justification for the number of clusters using heuristic evaluation methods. (**A**) Top-3 representative clustering results from the grid search using DBSCAN, ordered by the number of clusters and sorted by silhouette score. (**B**) Bar graph showing the average silhouette score for each number of clusters using DBSCAN. Each dot represents one clustering result from the grid search. Clustering with three clusters yields a significantly higher silhouette score compared to other cluster counts. Unpaired t-test with Bonferroni correction. (**C**) Same as (A), but clustering labels are assigned using K-means. (**D**) Bar graph showing the average inertia for different numbers of clusters using K-means. This follows the heuristic elbow method to justify the optimal cluster number. K-means requires a pre-specified number of clusters, and three clusters correspond to the "elbow point" based on the change in slope across different cluster counts. (**E**) Same as (A), but clustering labels are assigned using spectral clustering. (**F**) Bar graph showing eigenvalues corresponding to different pre-specified cluster numbers, calculated from the affinity matrix. This follows the eigen-gap heuristic, where a large gap between successive eigenvalues suggests the optimal number of clusters, again indicating three as a robust choice.

Figure S22. Classifier applied on PSE dataset predicts enhancement in both putative SST-Calb2 and SST-O cells

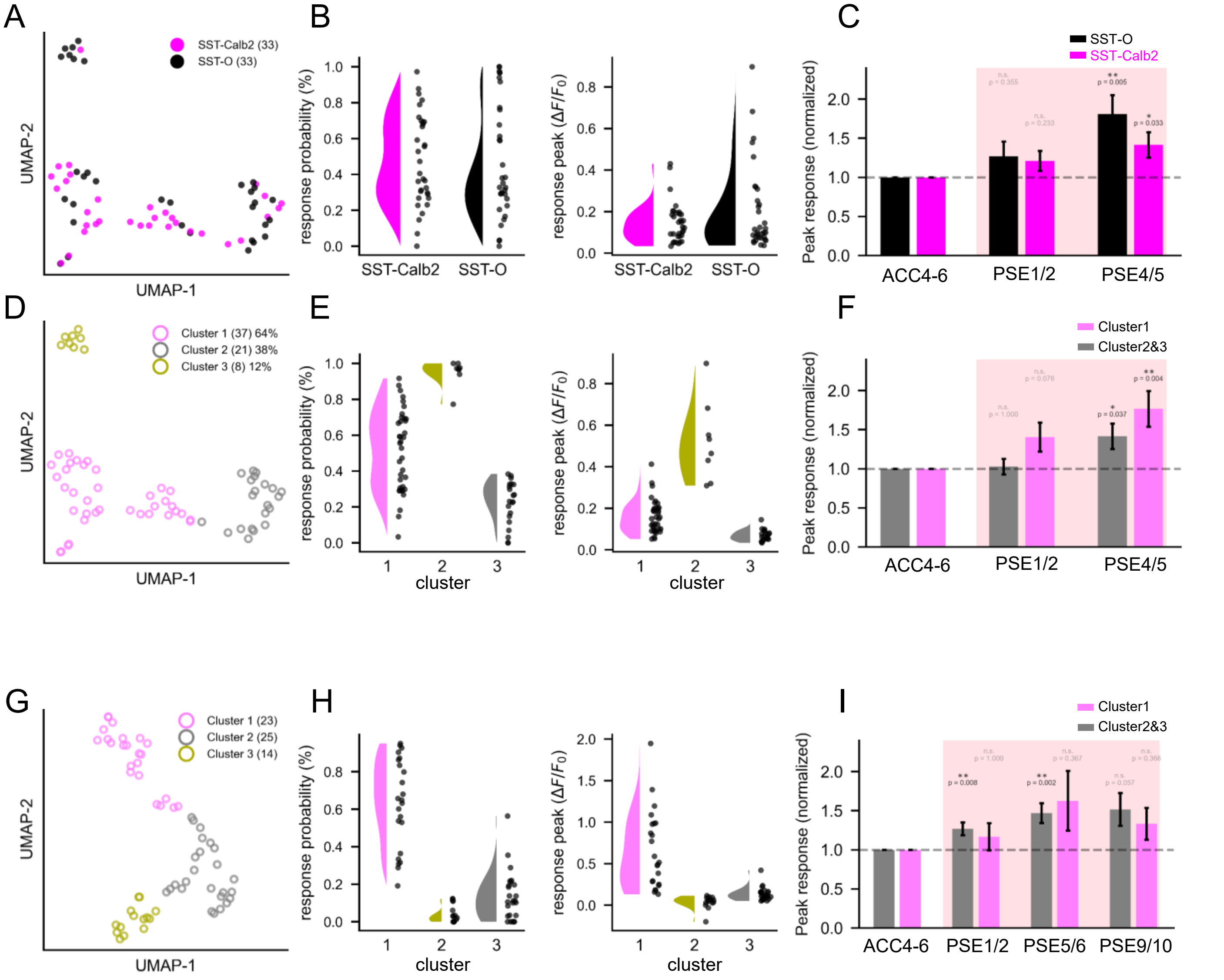

Fig. S22. Classifier applied on PSE dataset predicts enhancement in both putative SST-Calb2 and SST-O cells. (**A**) UMAP visualization of SST neuron clustering using response properties for ACC4-6 (n=66 cells in 6 mice) in SST-Flp x Calb2-Cre PSE dataset. (**B**) Example feature comparison of stimulus-evoked response probability for genetically-labeled SST-Calb2 and SST-O neurons. The evoked response probability and amplitude show distinct distributions in the two groups. (**C**) SST-Calb2 neurons show enhancement in mean peak evoked-response (all trials) during PSE, normalized to ACC4-6. Data averaged for PSE1/2 and PSE4/5. Paired t-test with Bonferroni correction, comparing ACC4-6 with training days. (**D**) Clustering assignment of putative SST-Calb2 (24/37, 64% SST-Calb2 cells in cluster 1) and putative SST-O neurons (8/21, 38% in cluster 2; 1/8, 12% in cluster 3) from the UMAP visualization in (A). (**E**) As in (B) but for the assigned clusters. (**F**) As in (C), but for putative SST-Calb2 neurons (cluster 1) versus cluster 2&3 (combined for comparison due to the small number of cell in cluster 3). Both putative SST-Calb2 (cluster 1) and SST-O (cluster 2&3) show enhancement in mean peak of stimulus-evoked response during PSE, normalized to ACC4-6. Paired t-test with Bonferroni correction. (**G**) As in (D) but for the unlabeled PSE dataset, with putative groups identified by clustering (n=62 cells in 5 mice). The putative SST-Calb2 group (cluster 1, 23/62 cells) and putative SST-O (cluster 2, 25/62 cells; cluster 3,14/62 cells) were identified. (**H**) As in (E), but for clusters identified in the unlabeled PSE dataset. (**I**) As in (F), but for putative SST-Calb2 (cluster 1) and combined cluster 2&3 from the unlabeled PSE dataset. Paired t-test with Bonferroni correction.

Table 1. Response Probability Features

Parameters	***Options***	***Description***
Trial type	Stimulus trials	Calculates response probability using only stimulus trials.
	Blank trials	Calculates response probability using only blank trials.
	All trials	Calculates response probability using all trial types.
In-trial periods	Pre-trial period	Uses peak signal from -2 to 0 seconds relative to trial onset to test against threshold.
	Evoked-trial period	Uses peak signal from 1 to 2 seconds after trial onset to test against threshold.
	Post-trial period	Uses peak signal from 2 to 4 seconds after trial onset to test against threshold.
Threshold	$1\sigma_1$, $2\sigma_1$, $3\sigma_1$, $5\sigma_1$, $10\sigma_1$	A trial is marked as responsive if its peak exceeds the selected threshold. Thresholds are multiples of σ_1, the standard deviation of the concatenated baseline period (1s window before trial onset) across all trials in the session.

Table 2. In-trial Measurement Features

Parameters	***Options***	***Description***	
Trial type	Stimulus trials	Calculates metric using only stimulus trials.	
	Blank trials	Calculates metric using only blank trials.	
	All trials	Calculates metric using all trial types.	
In-trial periods	Pre-trial period	Uses signal from -2 to 0 seconds relative to trial onset to calculate metric.	
	Evoked-trial period	Uses signal from 1 to 2 seconds after trial onset to calculate metric.	
	Post-trial period	Uses signal from 2 to 4 seconds after trial onset to calculate metric.	
Metrics	Peak	The largest $\Delta F/F_0$ value in the selected signal.	Unit: $\Delta F/F_0$
	Peak latency (timing)	The time offset of the largest $\Delta F/F_0$ value from trial onset in the selected signal.	Unit: s
	center of mass	The time offset of the center of the mass from trial onset in the selected signal. The COM is calculated by weighted integration along the time axis.	Unit: s
	area under the curve	The area under the curve is also calculated by integrating along the time axis using the composite trapezoidal rule	Unit: $\Delta F/F_0 \times s$

Table 3. Spontaneous Activity Measurement Features

Parameters	***Options***	***Description***	
Spontaneous block type	Initial block	Calculates metric using the 100s spontaneous block before task start.	
	Final block	Calculates metric using the 100s spontaneous block after task end.	
	Inter-trial blocks	Calculates metric using each inter-trial block starting from 5s after the previous trial ends to 3s before the next trial starts.	
	Initial&final block	Calculates the metric using both the initial block and final block.	
Threshold	$1\sigma_2$, $2\sigma_2$, $5\sigma_2$, $10\sigma_2$	Spontaneous events are detected if its prominence exceeds the selected threshold. Thresholds are multiples of σ_2, the standard deviation of the 20th percentile filter detrended (sliding window of 1 minute) fluorescence traces from whole session.	
Metrics	Peak	The average peak amplitude of all detected peaks.	Unit: $\Delta F/F_0$
	Scale (#event)	Number of peaks detected	Unit: #
	area under the curve	The area under the curve is calculated by integrating along the time axis using the composite trapezoidal rule.	Unit: $\Delta F/F_0 \times s$
